# Catalytic and structural properties of ATP‐dependent caprolactamase from *Pseudomonas jessenii*

**DOI:** 10.1002/prot.26082

**Published:** 2021-05-06

**Authors:** Antonija Marjanovic, Henriëtte J. Rozeboom, Meintje S. de Vries, Clemens Mayer, Marleen Otzen, Hein J. Wijma, Dick B. Janssen

**Affiliations:** ^1^ Biotransformation and Biocatalysis, Groningen Biomolecular Sciences and Biotechnology Institute (GBB) University of Groningen Groningen The Netherlands; ^2^ Biomolecular Chemistry and Catalysis, Stratingh Institute for Chemistry University of Groningen Groningen The Netherlands

**Keywords:** 5‐oxoproline, 6‐aminocaproic acid, carboxylase, hydrolase, lactamase, nylon 6, phosphorylation

## Abstract

Caprolactamase is the first enzyme in the caprolactam degradation pathway of *Pseudomonas jessenii*. It is composed of two subunits (CapA and CapB) and sequence‐related to other ATP‐dependent enzymes involved in lactam hydrolysis, like 5‐oxoprolinases and hydantoinases. Low sequence similarity also exists with ATP‐dependent acetone‐ and acetophenone carboxylases. The caprolactamase was produced in *Escherichia coli*, isolated by His‐tag affinity chromatography, and subjected to functional and structural studies. Activity toward caprolactam required ATP and was dependent on the presence of bicarbonate in the assay buffer. The hydrolysis product was identified as 6‐aminocaproic acid. Quantum mechanical modeling indicated that the hydrolysis of caprolactam was highly disfavored (ΔG_0_'= 23 kJ/mol), which explained the ATP dependence. A crystal structure showed that the enzyme exists as an (αβ)_2_ tetramer and revealed an ATP‐binding site in CapA and a Zn‐coordinating site in CapB. Mutations in the ATP‐binding site of CapA (D11A and D295A) significantly reduced product formation. Mutants with substitutions in the metal binding site of CapB (D41A, H99A, D101A, and H124A) were inactive and less thermostable than the wild‐type enzyme. These residues proved to be essential for activity and on basis of the experimental findings we propose possible mechanisms for ATP‐dependent lactam hydrolysis.

## 1 INTRODUCTION

ε‐Caprolactam is used as the building block for the homopolymer nylon‐6. Non‐reacted caprolactam may be discharged via wastewater at nylon‐6 production plants. Release of high levels of caprolactam in the environment should be avoided since it can be neurotoxic in various mammalian species as well as have phytotoxic effects.^1^ It has limited acute toxicity to humans and it is rapidly eliminated from the body.^1^, ^2^ Biodegradation of caprolactam is important for wastewater treatment at nylon production plants as well as for the removal of caprolactam that passes treatment systems or that is accidentally released into the environment.

**FIGURE 1:**
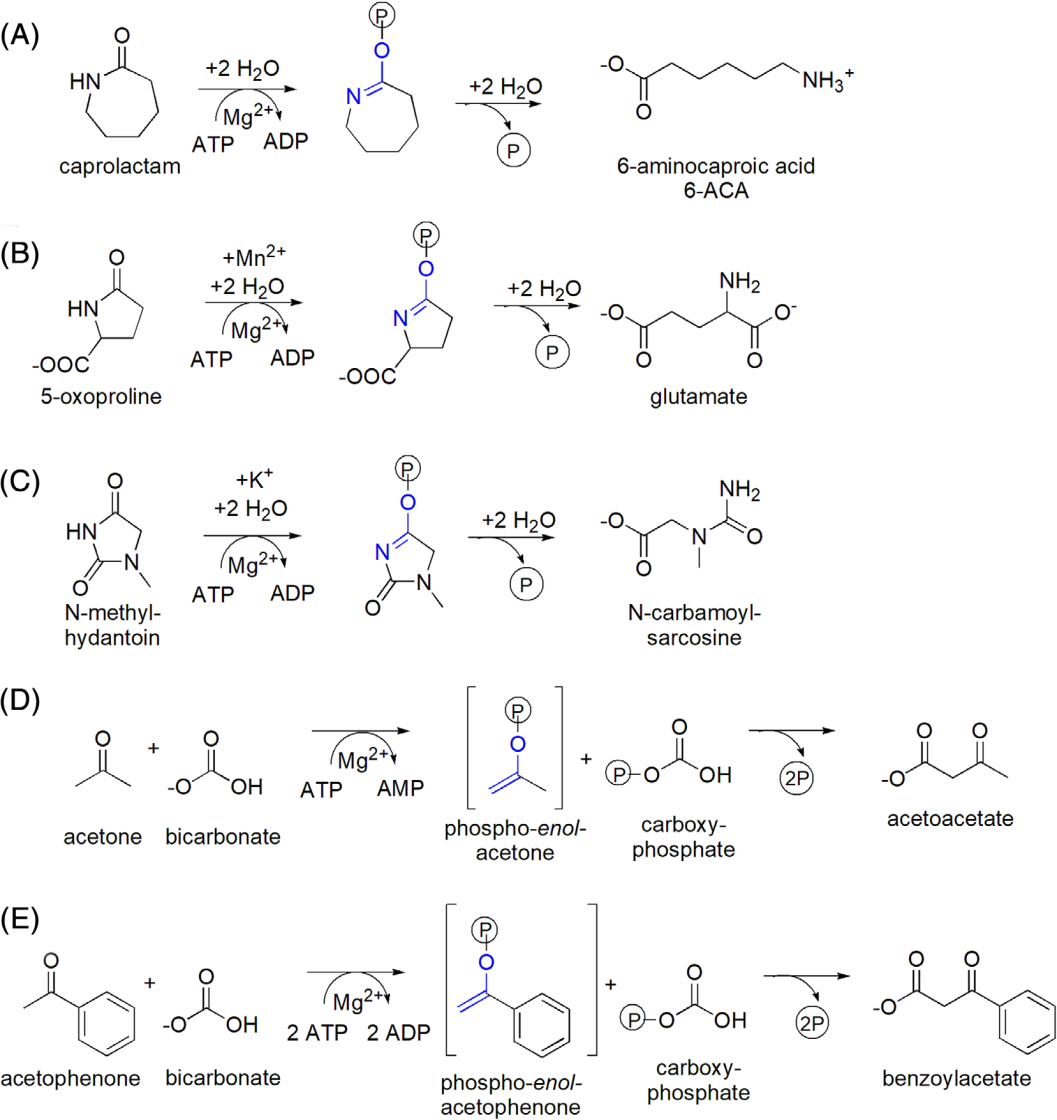
Reactions catalyzed by sequence‐related ATP‐dependent hydrolases/carboxylases. The reactions of caprolactamase, A; 5‐oxoprolinase,^12^ B; and hydantoinase,^13^ C; consume one equivalent of ATP. Acetone carboxylase,^14^ D; and acetophenone carboxylase,^15^ E perform a nucleophilic addition reaction between a phospho‐enol intermediate and carboxyphosphate using two phosphates from either one ATP molecule producing AMP, D or from two distinct ones producing ADP, E [Color figure can be viewed at wileyonlinelibrary.com]

Several microorganisms are known to degrade caprolactam under aerobic conditions, including strains of *Alcaligenes faecalis*, *Achromobacter guttatus* and different pseudomonads that use it as a growth substrate.^3^, ^4^, ^5^, ^6^, ^7^, ^8^ A caprolactam degradation pathway in these organisms was proposed already in the 1980s.^9^, ^10^ Conversion was supposed to start with lactam ring‐opening, followed by transamination and β‐oxidation of the 6‐oxo fatty acid. Whereas plasmids encoding caprolactam degradation genes were discovered in *Pseudomonas putida*,^10^, ^11^ the enzymes involved were not characterized until we recently described the pathway in the caprolactam‐utilizing bacterium *P. jessenii* strain GO3.^6^ Proteomics analysis indicated that cells growing on caprolactam produced large amounts of two different polypeptides with sequence similarity to eukaryotic oxoprolinases. Activity assays with lysates of *Escherichia coli* cells producing these proteins indicated they are subunits of a caprolactamase (EC 3.5.2.‐x) that catalyzes the first step in the catabolic pathway: the ATP‐dependent hydrolysis of the lactam ring to form 6‐aminocaproic acid (6‐ACA) (Figure 1). Next, 6‐ACA is deaminated to the aldehyde 6‐oxohexanoic acid by a transaminase.^16^ The aldehyde is oxidized to adipic acid by a dehydrogenase and subsequently metabolized in a β‐oxidation pathway. In *P. jessenii* GO3, the transaminase and β‐oxidation enzymes are also upregulated in cells growing on caprolactam as carbon and nitrogen source in comparison to glucose‐grown cells.^6^

The caprolactamase of *P. jessenii* GO3 is encoded by the *capA* (ORF CRX42_01175) and *capB* (CRX42_01180) genes, which are located in a single operon. Sequence analysis placed the CapAB enzyme in the hydantoinase/oxoprolinase family (Pfam CL0108). These enzymes are related to the actin‐like ATPase superfamily and ATP‐dependent lactamases/carboxylases. Homologs of caprolactamase include mammalian 5‐oxoprolinases and bacterial hydantoinases, but also acetone carboxylases and acetophenone carboxylase.^6^, ^17^, ^18^ Like caprolactamase, hydantoinase and 5‐oxoprolinase catalyze ATP‐dependent hydrolytic lactam ring opening (Figure 1A‐C).^12^, ^13^ CapAB has no 5‐oxoprolinase activity and to our knowledge it is the only enzyme of this group that is shown to convert caprolactam. The reaction catalyzed by the carboxylases is seemingly very different, that is, C–C bond formation instead of C–N bond hydrolysis (Figure 1D,E). Whereas the lactam hydrolysis reaction is accompanied by cleavage of a single high‐energy phosphoester bond, the carboxylation reactions require two, either by converting 1 ATP to 1 AMP and 2 phosphate (Figure 1D) or by hydrolyzing 2 ATP to 2 ADP and 2 phosphate (Figure 1E).

The ATP‐dependence of carboxylase reactions is not surprising from a thermodynamic point of view, but the ATP requirement of lactam hydrolysis is less obvious since most amide and lactam hydrolysis reactions are exergonic.^19^, ^20^ Whether this is also the case for caprolactam is unclear, but hydrolytic cleavage of L‐α‐amino‐ε‐caprolactamase has been reported with no mentioning of ATP dependence.^21^ Furthermore, an oxoprolinase from *A. faecalis* was ATP‐independent and reversible, with the equilibrium toward the lactam instead of the ring‐opened product.^22^ Peptide bond hydrolysis can also be ATP dependent. Here ATP plays a role in conformational changes in large protein assemblies and stimulates substrate binding.^23^, ^24^

Thus far, no structures are known for ATP‐dependent lactamases, hydantoinases, oxoprolinases or any other member of the oxoprolinase/hydantoinase family of ATP‐dependent hydrolases. Furthermore, mechanistic information is scarce. It has been proposed that hydrolysis of 5‐oxoproline proceeds via phosphorylation of the iminol tautomer in the large subunit and lactam cleavage in the other subunit.^25^ Crystal structures of acetone carboxylase from *Xanthobacter autotrophicus* (*Xa*Ac, PDB) and acetophenone carboxylase from *Aromatoleum aromaticum* (*Aa*Apc, PDB) were recently published^14^, ^15^ and suggested that different subunits are involved in ATP‐dependent substrate phosphorylation and carbon‐carbon bond formation. To understand the relation between ATP‐dependent carboxylase and lactamase enzymes, we analyzed the sequence and solved the crystal structure of the CapAB protein. Comparison to carboxylase structures provided information on the function of the CapAB subunits and the active site residues. Accordingly, mutants of CapAB carrying substitutions in conserved motifs were constructed. The activities of the mutants and observation that caprolactam hydrolysis was dependent on the presence of bicarbonate suggested possible mechanisms for ATP‐dependent lactam hydrolysis.

## 2 RESULTS AND DISCUSSION

### 2.1 Bioinformatic analysis

Proteomic‐ and sequence analysis of the *P. jessenii* GO3 genome led to the discovery of the *capAB* genes which encode polypeptides of 696 (CapA, UniProtKB A0A2W0EVE0, 75 kDa) and 581 amino acids (CapB, UniProtKB A0A2W0FH34, 63 kDa). The proteins were upregulated in cells growing on caprolactam as compared to cells cultivated on glucose. Among known proteins with confirmed activity, the closest homologs of caprolactamase are eukaryotic oxoprolinases (OP),^6^ for example the human, bovine and rat enzymes and the oxoprolinase from *Saccharomyces cerevisiae*.^26^, ^27^, ^28^, ^29^ These are dimeric enzymes with subunits of around 1250 amino acids. Sequence alignments showed that CapA is similar to the N‐terminal part of ca. 730 amino acids of the oxoprolinase protein, whereas CapB is homologous to the C‐terminal part of ca. 550 amino acids (Table 1). Obviously, the subunit composition of enzymes in the ATP‐dependent lactamases and carboxylases family varies, with the oxoprolinases corresponding to the fused lactamase subunits. To comply with the terminology proposed by Weidenweber et al^15^ for acetone carboxylase (see below) we call the expected ATP‐binding subunit of caprolactamase subunit α or CapA and the other (smaller) subunit β or CapB; these align to the oxoprolinase sequence in the order of A‐B, but are encoded on the *P. jessenii* genome and in the expression clone in the order of B‐A.

**TABLE 1:** Sequence similarities of CapAB

PjCapA and homologs	Source	aa/segment	% id.	Accession (UniProt/PDB)	Ref.
Caprolactamase (PjCapA)	*Pseudomonas jessenii* GO3	696 aa	100	A0A2W0EVE0	6
Oxoprolinase (BtOplA)	*Bos taurus*	1‐724		Q75WB5	27
Oxoprolinase (HsOplA)	*Homo sapiens*	1–724	29	O14841	26
Oxoprolinase (RnOplA)	*Rattus norvegicus*	1–724		P97608	28
Oxoprolinase (ScOplA)	*Saccharomyces cerevisiae*	1‐732	26	P28273	30
Acetophenone carboxylase γ subunit (*Aa*Apcα)^a^	*Aromatoleum aromaticum* EbN1	732 aa	33	Q5P5G4, 5L9W_B	15
Acetophenone carboxylase α subunit (*Aa*Apcα′)	*Aromatoleum aromaticum* EbN1	658 aa	27	Q5P5G2, 5L9W_b	15
Acetone carboxylase β subunit (*Xa*Acβ)^b^	*Xanthobacter autotrophicus* Py2	717 aa	26	Q8RM04, 5SVB_B	18
Hydantoinase‐like hydrolase IaaCE (OaHyuA)	*Aromatoleum aromaticum*	707 aa	37	Q5P602	31
5‐substituted hydantoinase (PspHyuA)	*Pseudomonas sp*. strain NS671	690 aa	32	Q01262	32
Lactamase (PpLactA)	*Pseudomonas putida* KT2440	694 aa	80	Q88H50	33
Match with N‐terminal sequence of the large subunit of N‐methylhydantoin hydrolase from *Pseudomonas putida* 77	*Paracoccus denitrificans* strain 1222	692 aa	32	Q9R4N3/A1BA73	34
**PjCapB and homologs**					
Caprolactamase (PjCapB)	*Pseudomonas jessenii* GO3	581 aa	100	A0A2W0FH34	6
Oxoprolinase (BtOplA)	*Bos taurus*	730‐1288		Q75WB5	27
Oxoprolinase (HsOplA)	*Homo sapiens*	728‐1256	27	O14841	26
Oxoprolinase (RnOplA)	*Rattus norvegicus*	730–1288	27	P97608	28
Oxoprolinase (ScOplA)	*Saccharomyces cerevisiae*	741‐1282	26	P28273	30
Acetophenone carboxylase β subunit (*Aa*Apcβ)	*Aromatoleum aromaticum* EbN1	684 aa 26‐624	23	Q5P5G3, 5L9W_C	15
Acetone carboxylase α subunit (*Xa*Acα)^b^	*Xanthobacter autotrophicus* Py2	776 aa (47‐666)	19	Q8RM03, 5M45_A	18
Hydantoinase‐like hydrolase IaaCE (AaHyuB)	*Aromatoleum aromaticum*	564 aa	36	Q5P600	31
5‐substituted hydantoinase (PspHyuB)	*Pseudomonas sp*. strain NS671	583 aa	31	Q01263	32
Lactamase (PpLactB)	*Pseudomonas putida* KT2440	581 aa	86	Q88H51	33
Match with N‐terminal sequence of the small subunit of N‐methylhydantoin hydrolase from *Pseudomonas putida* 77	*Paracoccus denitrificans* strain 1222	594 aa	30	Q9R4N2/ A1BA72	34

^a^ Annotated as gamma subunit in 5L9W_B.

^b^ According to the unified terminology by Weidenweber et al,^15^ the subunit names of XaAc should be reversed.

Searching genomic databases shows that *capAB* genes and close homologs are not rare. Highly similar coding sequences (80%‐98% identity) occur in many *Pseudomonas* strains, with the encoded proteins annotated as hydantoinase/oxoprolinase family enzymes. Since for many of these the sequence similarity to the *P. jessenii* caprolactamase is much higher than to confirmed oxoprolinases or hydantoinases, the genes more likely encode lactamases. However, the function of these genes or proteins has not been established, with the exception of the *oplAB* genes of *P. putida* KT2440.^35^ These *oplAB* genes were upregulated in cultures growing in the presence of valerolactam and caprolactam, and gene knockouts confirmed their role in lactam utilization. Yet, no activity was found when these genes, which encode proteins that share 80% to 86% sequence identity to CapAB, were expressed in *E. coli*.^33^, ^35^

Whereas CapAB is clearly related to eukaryotic oxoprolinases, sequence comparison showed that caprolactamase is not similar to a group of prokaryotic 5‐oxoprolinases recently identified by Niehaus et al.^36^ Bioinformatic analysis showed that various bacterial genomes harbor *pxpABC* genes that are essential for ring opening of 5‐oxoproline formed from glutamic acid residues during in vivo peptide degradation. The PxpA protein is probably a metal‐dependent tetramer with lactamase activity while the co‐purified PxpB and PxpC proteins show ATP hydrolysis activity.^37^ This class of oxoprolinases may be related to the two‐component enzyme converting oxoproline to glutamate in *P. putida*.^38^, ^39^, ^40^ Component A is a hexamer of dimers (64 kDa and 51 kDa polypeptides) that catalyzes oxoproline‐dependent ATP hydrolysis and component B (82 kDa) is required for 5‐oxoproline amide bond hydrolysis. PxpABC also shows no similarity to ATP‐dependent carboxylases.

Similar to caprolactamase, the homologous ATP‐dependent hydantoinases are composed of unfused smaller subunits (Table 1), unlike the oxoprolinases. *Pseudomonas sp*. strain NS671 harbors three plasmid‐localized genes (*hyuABC*) which encode a non‐stereospecific hydantoinase. The gene products HyuAB hydrolyse 5‐substituted hydantoins to the corresponding N‐carbamyl amino acids, whereas HyuC converts the N‐carbamyl amino acids into free amino acids.^32^ The oligomeric structure of this hydantoinase is not known, but the N‐methyl‐hydantoinase from *P. putida* 77 is a heterotetramer consisting of two large and two small subunits.^34^ The reported N‐terminal sequences of the subunits indicate sequence similarity to proteins of the hydantoinase/oxoprolinase family, including the CapAB protein described here (Table 1). Additionally, another homolog is found in the metabolism of indolacetate found in the bacteria *A. aromaticum* and *Azoarcus evansii*.^31^ The hydrolysis of the N‐heterocyclic ring of 2‐oxoindolacetate was proposed to be catalyzed by a hydantoinase‐like hydrolase in an ATP‐dependent reaction. The *iaaCE* genes for this heteromeric enzyme are part of a gene cluster which is upregulated when the bacteria are grown anaerobically on indolacetate as carbon and energy source.

Of the caprolactamase homologs, crystal structures have only been solved for two carboxylases. The structure of acetophenone carboxylase from *A. aromaticum* EbN1 (*Aa*Apc) was reported by Weidenweber et al.^15^ (PDB‐ID: 5L9W), and the structure of acetone carboxylase from *X. autotrophicus* (*Xa*Ac) was determined in multiple conformational states by Mus et al^14^ (PDB‐ID: 5 M45, 5SVB, 5SVC). These enzymes have more complex quaternary structures than CapAB and related lactamases. *Xa*Ac occurs as a heterohexamer that consists of three different subunits: (αβγ)_2_. *Aa*Apc consists of a heterooctameric core complex (αα′βγ)_2_ and a smaller subunit Apcε which dissociates from the core complex during purification but is required for activity.^41^

**FIGURE 2:**
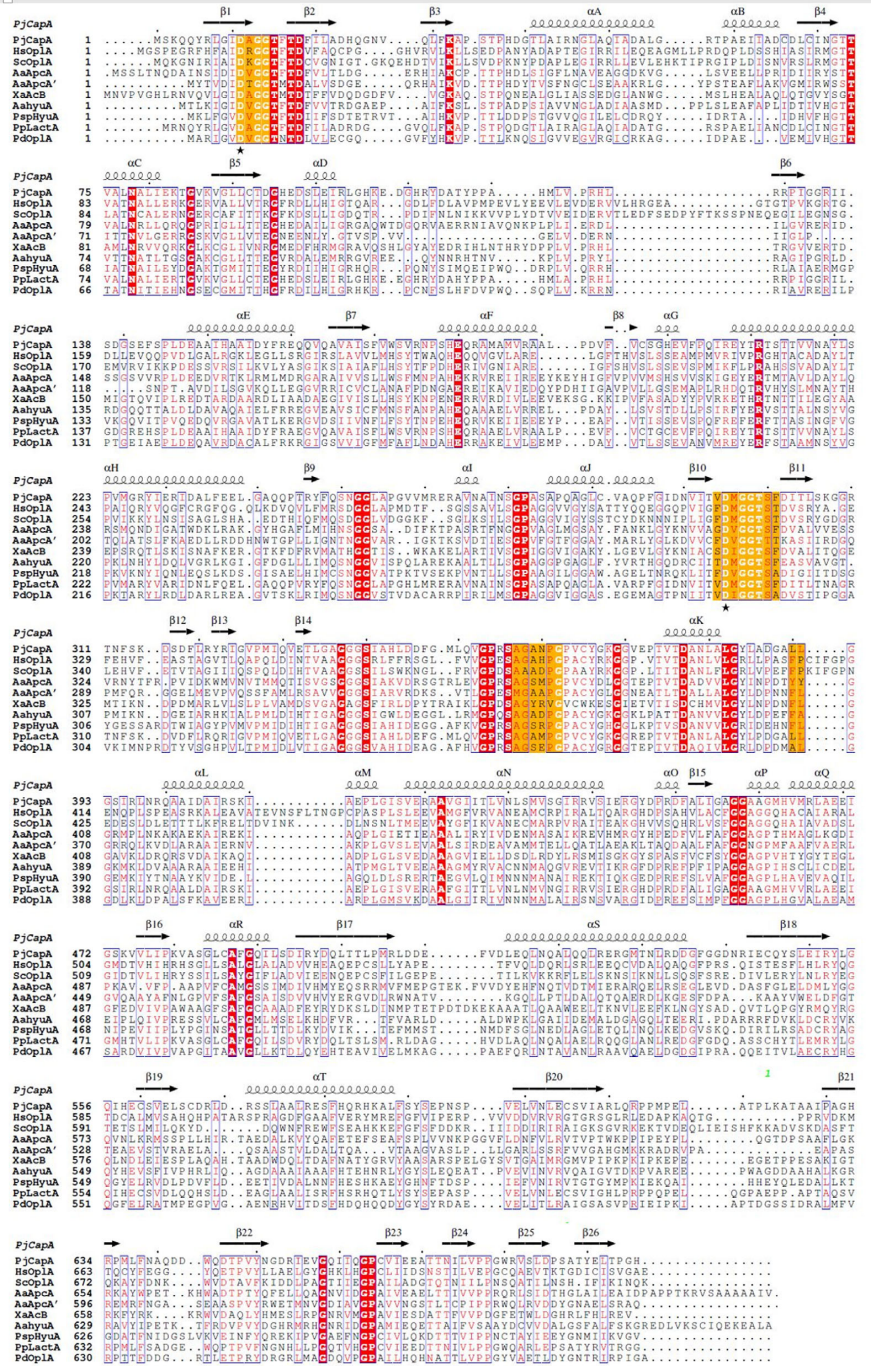
Multiple sequence alignment of CapA with related ATP‐dependent hydrolases/carboxylases. Sequences: PjCapA, subunit A of caprolactamase from *P*seudomonas *jessenii* GO3; HsOplA, N‐terminal part of 5‐oxoprolinase from *Homo sapiens*; ScOplA, idem, from *Sacchharomyces cerevisiae*; AaApcA and A′, acetophenone carboxylase from *Aromatoleum aromaticum*; *Xa*AcB, acetone carboxylase from *Xanthobacter autotrophicus*; AaHyuA, hydantoinase‐like hydrolase IaaCE from *A. aromaticum*; PspHyuA, hydantoinase from *Pseudomonas* sp. strain NS671; PpLactA, lactamase from *Pseudomonas putida* KT2440; PdOplA, 5‐oxoprolinase from *Paracoccus denitrificans* strain 1222. The secondary structure features are taken from the crystal structure of PjCapA (vide infra). P1: γ‐phosphate binding site (DxGGTxTD). P2: β‐phosphate binding site (DxGGT). The first aspartic acids from the two phosphate binding sites were chosen for mutagenesis (asterisk). A: Adenosine binding site (GxxPGP). The figure was created with ESPript^42^ [Color figure can be viewed at wileyonlinelibrary.com]

CapA is homologous to the *Xa*Acβ subunit and to both the *α* and α′ subunit of *Aa*Apcα (Table 1). These carboxylase subunits are involved in substrate phosphorylation. The *Xa*Acβ subunit, *Aa*Apcα and *Aa*Apcα′ are structurally related and all three possess an ATP binding site from the ASKHA superfamily (acetate and sugar kinase/hsp70/actin). Multiple sequence alignment of CapA with the homologous ATP‐binding carboxylase subunits and annotated sequences of related hydantoinase/oxoprolinase proteins revealed that several functional motifs are conserved (Figure 2). The sequence motifs for adenosine binding (GxxPGP) (G**AN**PGP, res. 358‐363 in CapA) and for binding of phosphate 1 (DxGGTxDDT) (D**A**GGT**FT**D**F**, 11‐18 in CapA) and phosphate 2 (DVGGT) (D**M**GGT, 295‐299 in CapA) of ATP are conserved in the ATP binding site^15^ (Figure 2). In acetone carboxylase, the *Xa*Acβ subunit consumes ATP and converts it to AMP with phosphorylation of both bicarbonate and the enol tautomer of acetone. In case of acetophenone carboxylase, phosphorylation of acetophenone and bicarbonate occurs in the separate Apcα and Apcα′ subunits of *Aa*Apc, each at the expense of one ATP to ADP conversion.

**FIGURE 3:**
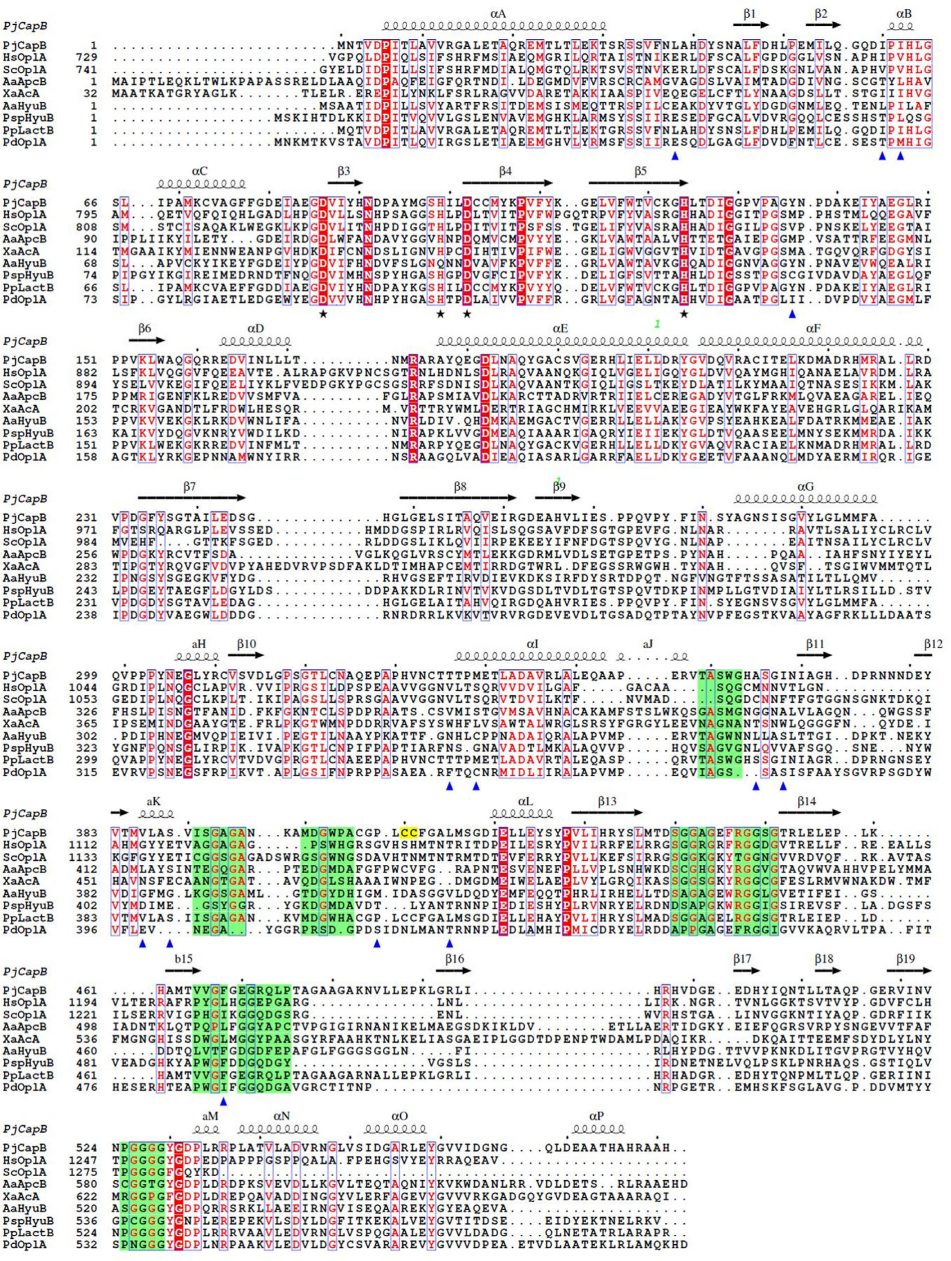
Multiple sequence alignment of CapB with related ATP‐dependent hydrolases/carboxylases. Sequences: PjCapB, caprolactamase from *Pseudomonas jessenii* GO3; HsOplA, C‐terminal part of 5‐oxoprolinase from *Homo sapiens*; ScOplC, idem, from *Saccharomyces cerevisiae*; AaApcB, acetophenone carboxylase β subunit from *Aromatoleum aromaticum*; XaAcA, acetone carboxylase from *Xanthobacter autotrophicus*; AaHyuB, hydantoinase‐like hydrolase IaaCE from *A. aromaticum*; PspHyuB, hydantoinase from *Pseudomonas* sp. strain NS671; PpLactB, lactamase from *Pseudomonas putida* KT2440; PdOplA, 5‐oxoprolinase from *Pseudomonas denitrificans* 1222. The secondary structure features are taken from the crystal structure of PjCapB (vide infra). Putative metal‐binding residues which are highly conserved among the examined sequences are marked with an asterisk. Blue triangles indicate residues in the active site. In yellow the vicinal disulfide is shown. The eight PG_II_ helical structural elements are depicted in green. The figure was created with ESPript^42^ [Color figure can be viewed at wileyonlinelibrary.com]

The smaller CapB subunit of caprolactamase is homologous to the *Xa*Acα subunit of acetone carboxylase and to the of *Aa*Apcβ subunit of acetophenone carboxylase (Table 2). These subunits contain a metal‐binding site and are involved in the carbonate coupling reaction, using phosphorylated bicarbonate as the activated donor.^14^, ^15^ In *Xa*Acα, residues Glu89, His150, Asp153, and His175 are involved in manganese binding. In the metal‐binding site of *Aa*Apcβ a non‐physiological mercury ion was found. It is liganded by Asp65, His123, Asp126, and His148. Of these carboxylase metal‐binding residues, the Asp‐His‐Asp‐His tetrad is conserved in CapB as Asp41, His99, Asp102, and His124 (Figure 3). From the sequence alignments, it appears that *Xa*Acα has Glu89 instead of the aspartate in the homologs. This glutamate is regarded to be a gating residue in the tunnel connecting the ATP‐dependent phosphorylation subunit with the metal‐containing coupling subunit.^14^

**TABLE 2:** Caprolactam and ATP hydrolysis activity of CapAB

	Reaction mixture^a^			Initial rate	
Entry	Cap (mM)	ATP (mM)	HCO_3_^−^ (mM)	ADP (U mg^−1^)	ACA (U mg^−1^)
1	2	2	50	0.51	0.39
2	0	2	50	0.44	—^b^
3	2	2	0	0.008	—
4	2	2	0.5	0.021	0.017
5	0	2	0.5	0.019	—
6	2	2	5	0.167	0.190
7	0	2	5	0.158	—
8^c^	2	0	50	—	—

^a^ Reaction mixtures contained CapAB (0.045 mg∙ml^−1^) and varying concentrations of ATP, caprolactam and bicarbonate in Hepes buffer, pH = 8.0.

^b^ In the absence of ATP no product formation was found (caprolactamase activity <0.004 U/mg).

^c^ Reference reaction from Otzen et al.^6^; reaction mixture containing CFE of CapAB producing *P. jessenii* strains grown on caprolactam.

The sequence motifs characteristic of the ATP binding site of CapA, *Xa*Acα, and *Aa*Apcβ are conserved in the oxoprolinase and hydantoinase sequences (Figure 2), including the lactamase of *P. putida* KT2440 (Table 1) that was recently proposed to be capable of caprolactam degradation.^35^ Furthermore the Asp/Glu‐His‐Asp‐His metal‐binding site in CapB is conserved in the related enzymes (Figure 3).

### 2.2 Expression and purification

**FIGURE 4:**
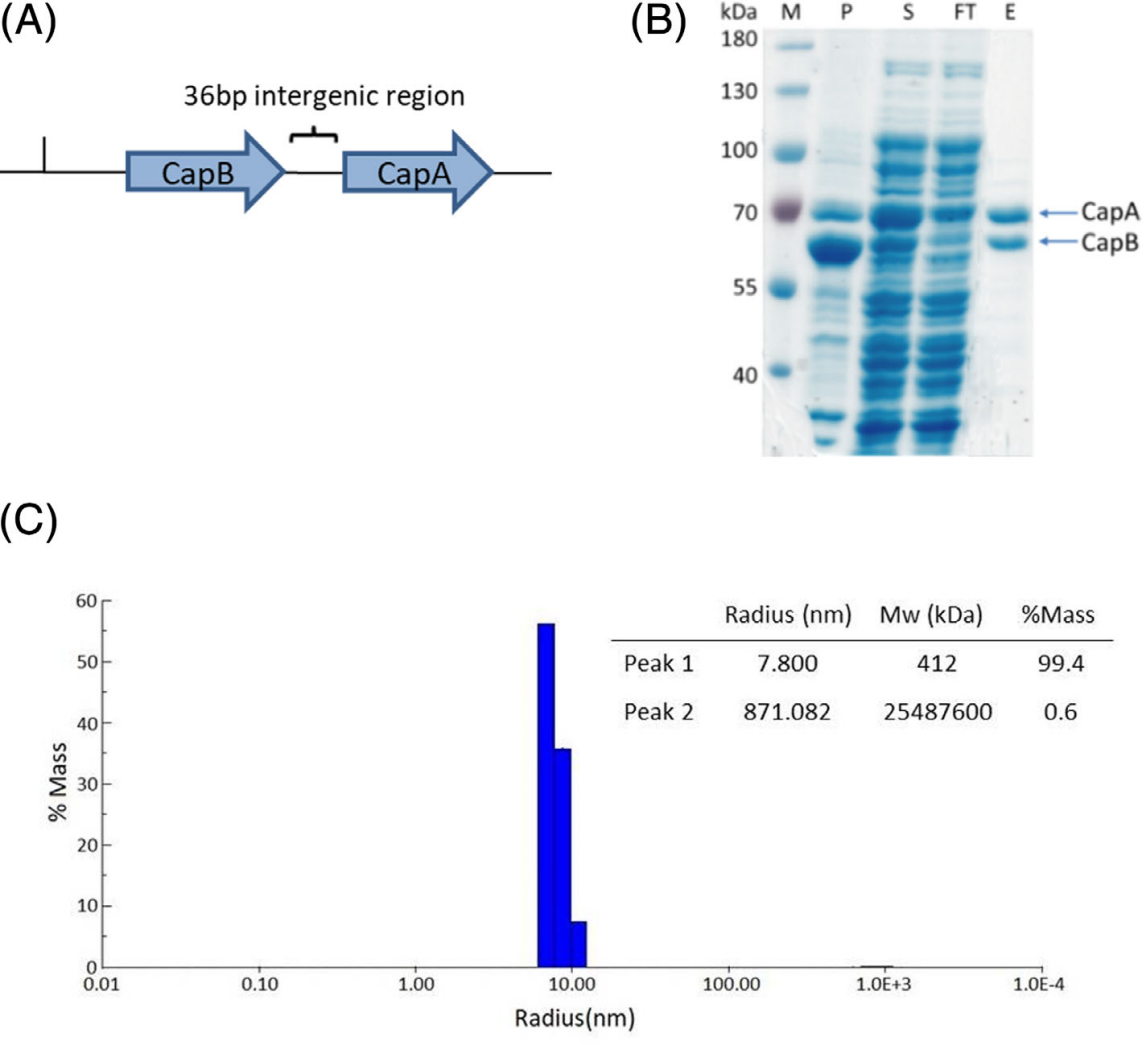
Expression and purification of CapAB. A, A 3.9 kb *Pseudomonas jessenii* DNA fragment harboring *capA* and *capB* genes was cloned behind the T7 promoter pET20(+) with a 36 bp intergenic region and a C‐terminal His tag coding sequence. B, SDS‐PAGE of the His‐tag purification of CapAB. M: Protein ladder; P: Pellet fraction after sonication; S: Supernatant fraction after sonication; FT: Unbound proteins after incubation of supernatant fraction with the Ni‐resin; E: Elution of bound protein. C, Dynamic light scattering (DLS) of purified caprolactamase. The majority (99.4% mass) of the protein belongs to the species with a radius of 7.8 nm. The molecular weight of the protein is overestimated due to its non‐globular assembly. Some aggregation is observed (0.6%) [Color figure can be viewed at wileyonlinelibrary.com]

To isolate and characterize caprolactamase we produced the protein in *E. coli* using a pET‐derived construct harboring the CapB‐CapA coding sequence amplified from *P. jessenii* genomic DNA (Figure 4A). The *capB‐capA* intergenic region from *P. jessenii* which includes a ribosome binding site for CapA expression was maintained. The C‐terminal end of CapA was fused to a poly‐histidine tag whereas CapB stayed untagged. CapAB was subsequently produced in *E. coli* C41(DE3) growing in TB medium. Analysis of centrifuged cell lysates by SDS‐PAGE showed that the CapA and CapB polypeptides were produced as soluble proteins, but a large amount of CapB also occurred as insoluble protein in the pellet (Figure 4B). The disproportional overexpression of CapB is likely due to the native *P. jessenii* ribosome binding site preceding *capA* having a lower ribosome recruitment rate.^43^ The CapAB protein was purified by metal affinity chromatography using a Ni‐NTA resin in a single purification step. Due to the tight interaction of the heterodimer both subunits were purified simultaneously via the CapA His‐tag. The yield of isolated CapAB protein was 50 mg/L culture. For crystallographic experiments CapAB was additionally purified by gel permeation chromatography.

### 2.3 Catalytic activity

**FIGURE 5:**
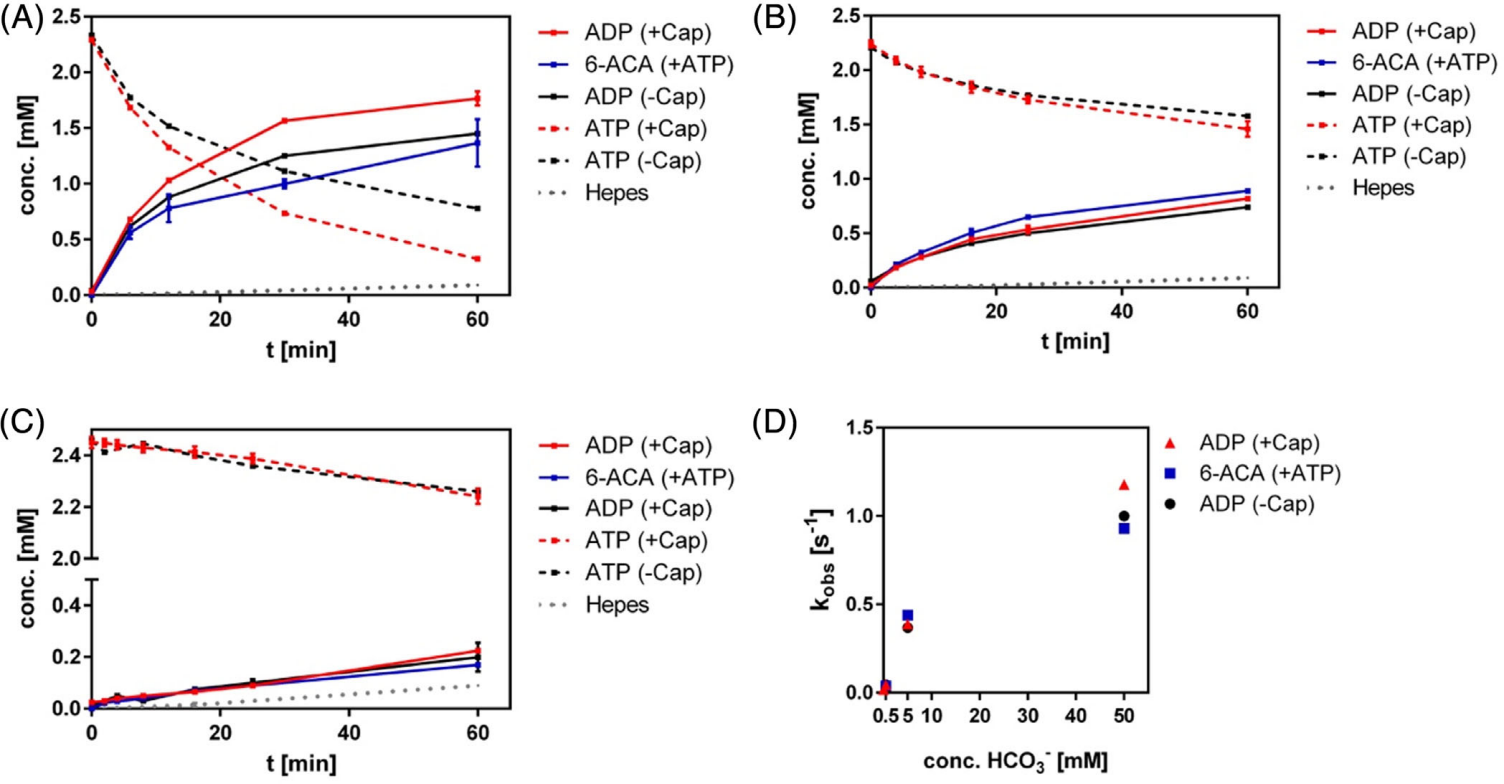
ATP and bicarbonate dependence of caprolactam hydrolysis. A, formation of 6‐ACA and ADP during caprolactam hydrolysis. Reaction mixtures contained 2 mM caprolactam, 2 mM ATP, 5 mM MgCl_2_ and 5% glycerol in 50 mM ammonium bicarbonate buffer, pH 8. Formation of ADP and reduction of ATP (red) was followed by HPLC. Formation of 6‐ACA (blue) was followed by UPLC/MS. Controls contained no caprolactam (black). Dotted line: [ADP] in Hepes only. B, ATP and caprolactam hydrolysis under conditions as in A, but with 5 instead of 50 mM ammonium bicarbonate. C, Idem, with 0.5 mM ammonium bicarbonate. D, Dependence of initial velocity of ATP and caprolactam hydrolysis on bicarbonate concentration, showing that both coupled (red, blue) and uncoupled hydrolysis (black) are bicarbonate dependent [Color figure can be viewed at wileyonlinelibrary.com]

To investigate the catalytic properties of caprolactamase we performed activity assays with purified enzyme. Hydrolysis of caprolactam to 6‐ACA by CapAB was dependent on the presence of ATP.^6^ The depletion of ATP and the formation of ADP were followed by HPLC and the caprolactam hydrolysis product 6‐ACA was quantified by UPLC‐MS. In the presence of caprolactam and ATP in bicarbonate buffer, the initial ACA formation activity was 0.51 U mg^−1^ (Table 2). Conversion with 2 mM ATP and 2 mM caprolactam leveled off at 1.4 mM ACA (Figure 5A). The molar ratio between hydrolysis of ATP to ADP and production of 6‐ACA was 1.2, suggesting some excess hydrolysis of ATP under these conditions, especially when the caprolactam concentration becomes lower as the reaction proceeds (Figure 5A).

If caprolactam was omitted, significant uncoupled hydrolysis of ATP was observed (Figure 5A). The rate of caprolactam‐independent ATP hydrolysis was ca. 70% of that observed with caprolactam present (Table 2, entries 2, 5, and 7). This high rate of uncoupling has been observed previously for N‐methylhydantoinase from *P. putida* 77 in the presence of some cyclic amide compounds that were not hydrolyzed themselves.^34^ Since in the presence of caprolactam production of 6‐ACA and ADP was almost in a stoichiometric ratio, caprolactam strongly suppressed the rate of uncoupled ATP hydrolysis. The high rate of uncoupling is surprising since the intracellular production of the CapAB under inducing conditions suggests the possibility of futile ATP hydrolysis.

To examine the role of bicarbonate in caprolactam and ATP hydrolysis, activity measurements were performed in Hepes buffer under the same conditions as described before. Omitting bicarbonate from the standard reaction mixture by using Hepes buffer reduced ATP hydrolysis in the presence of caprolactam by at least 60‐fold (Figure 5A). Furthermore, caprolactam hydrolysis was not detectable by 6‐ACA formation if bicarbonate was not present (Table 2). This lack of caprolactam hydrolysis in the absence of bicarbonate was not due to inhibition by Hepes because in the same buffer with 0.5 mM or 5 mM ammonium bicarbonate added the rate of ATP hydrolysis and 6‐ACA formation hydrolysis increased to the expected level (Figure 5B,C). In each case, the ATP hydrolysis activity found in the presence of caprolactam was only slightly higher than in its absence. These results show an essential role for bicarbonate in the hydrolysis of caprolactam and ATP and that the observed uncoupling was also dependent on the presence of HCO_3_^−^ (Figure 5D).

In a recent study on caprolactam degradation, Thompson et al.^33^ reported that expression in *E. coli* of CapAB homologs encoded by the *P. putida* KT2440 *oplAB* genes (Table 2) did not give detectable activity, but a possible effect of bicarbonate was not reported. Also earlier literature on hydantoinases and oxoprolinase activity do not report a requirement for bicarbonate in assay buffers.^32^, ^38^

### 2.4 Caprolactam hydrolysis is endergonic

**FIGURE 6:**
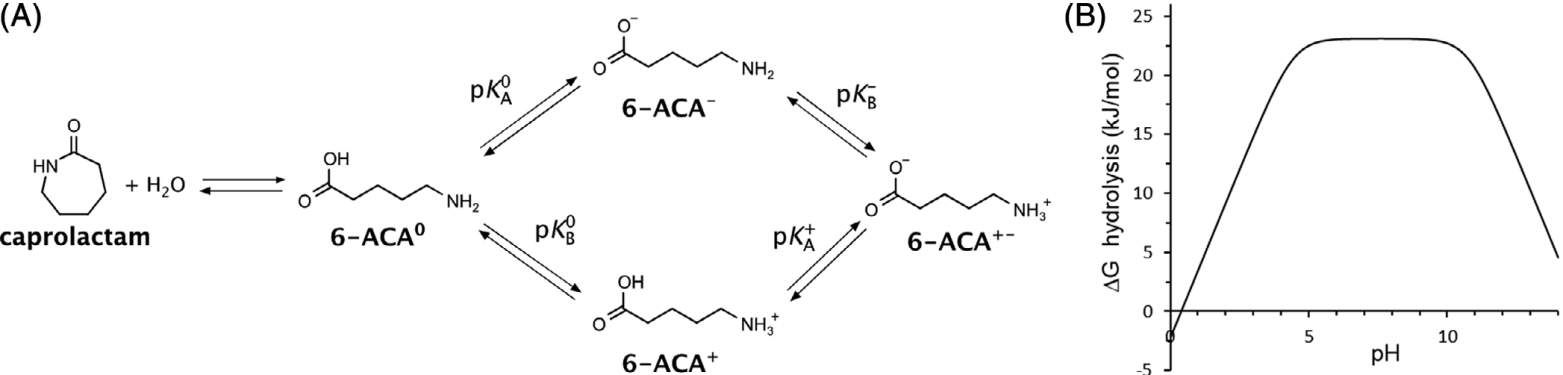
Energetics of caprolactam hydrolysis. A, Relevant species in the equilibrium between caprolactam and 6‐ACA. B, Predicted change in Gibbs free energy (ΔG_0_) upon hydrolysis of caprolactam to 6‐ACA (all species) in water. The difference in energy between the uncharged species (Scheme 1) was calculated quantum mechanically using CBS‐QB3 with an SMD solvent model,^44^.^45^ The energy differences with the charged 6‐ACA species were calculated from p*K*_A_ and p*K*_B_ constants (Materials and Methods [section 4])

The unexpected ATP dependence of caprolactam hydrolysis prompted us to calculate the free energy for this reaction. The equilibrium was computed under Gaussian using the CBS‐QB3 method, which can reproduce absolute formation energies with an error of <5 kJ/mol.^44^ The effect of pH was modeled from known acid and base dissociation constants (Figure 6A). Very acidic or basic conditions were predicted to make hydrolysis more favorable while at neutral pH the ring‐closed or lactam form is highly favored (Figure 6B). A similar pH dependence was experimentally observed for the uncatalyzed hydrolysis of 5‐oxoproline.^46^ The calculations showed that at neutral pH the caprolactam hydrolysis reaction was highly endergonic; the equilibrium disfavored formation of 6‐ACA (sum of all species) due to a Δ*G*_0_′ of 23 kJ/mol. By coupling the reaction to ATP hydrolysis (Δ*G*_0_′ = −31 kJ/mol^47^) the reaction becomes exergonic.

### 2.5 The structure of the CapAB dimer

A 4.0 Å resolution dataset was collected from a single crystal grown in phosphate‐containing Bis‐Tris propane buffer with 20% PEG3350. Extensive efforts to reproduce or obtain more or better diffracting crystals failed, also when substrate or substrate analogs were added. The structure was solved by molecular replacement and refined to an R/R_free_ of 29/37%. Despite the modest resolution of the data, the CapA and CapB main chains were well tracible and side chains of β‐strands and α‐helices can be observed in the electron density maps (Supporting Information Figures S1 and S2) and the structure was refined with reasonable validation statistics (Table 3). The Rama‐Z score of −3.3 is slightly better than the mean value of −4.0 for structures with similar resolution.^48^ The Molprobity score of 2.3 with a percentile of 99% implies that the actual crystallographic resolution is quality‐wise better than the average structure at that resolution (3.25‐4.25 Å).

**TABLE 3:** Data collection and refinement statistics

Data collection	
Data collection temperature	110 K
Unit cell a, c (Å)	195.4, 167.4, 88.0
Resolution (Å)	48.8‐4.0 (4.28‐4.0)
No. of observations	92 432 (15956)
No. of unique reflections	24 725 (4389)
R_pim_ (%)	37.0 (74.5)
Completeness (%)	98.8 (98.7)
Mean I/σ (I)	2.3 (1.1)
Redundancy	3.7 (3.6)
CC_(1/2)_	0.784 (0.389)
**Refinement**	
R/R_free_ (%)	29/37
No. of protein atoms	19 265
Protein B value (Å^2^) (chain A, B, C, D)	64.4, 54.8, 75.2, 59.7
Ligand active site	2 × Zn
Ligand B values (Å^2^) (zinc)	48.5 and 52.1
**Geometry**	
R.m.s. deviations, bond lengths (Å)	0.005
R.m.s. deviations, bond angles (^o^)	1.1
Ramachandran most favored (%)	89.34
Ramachandran allowed (%)	9.91
Ramachandran outliers (%)	0.75
Rama distribution Z‐score	−3.3
Rotamer outliers	0
Cβ outliers	0
MolProbity/percentile	2.3/99th
Clashscore/percentile	17.2/97th
PDB accession code	6YRA

*Note*: Numbers in parenthesis are for the highest resolution shell.

**FIGURE 7:**
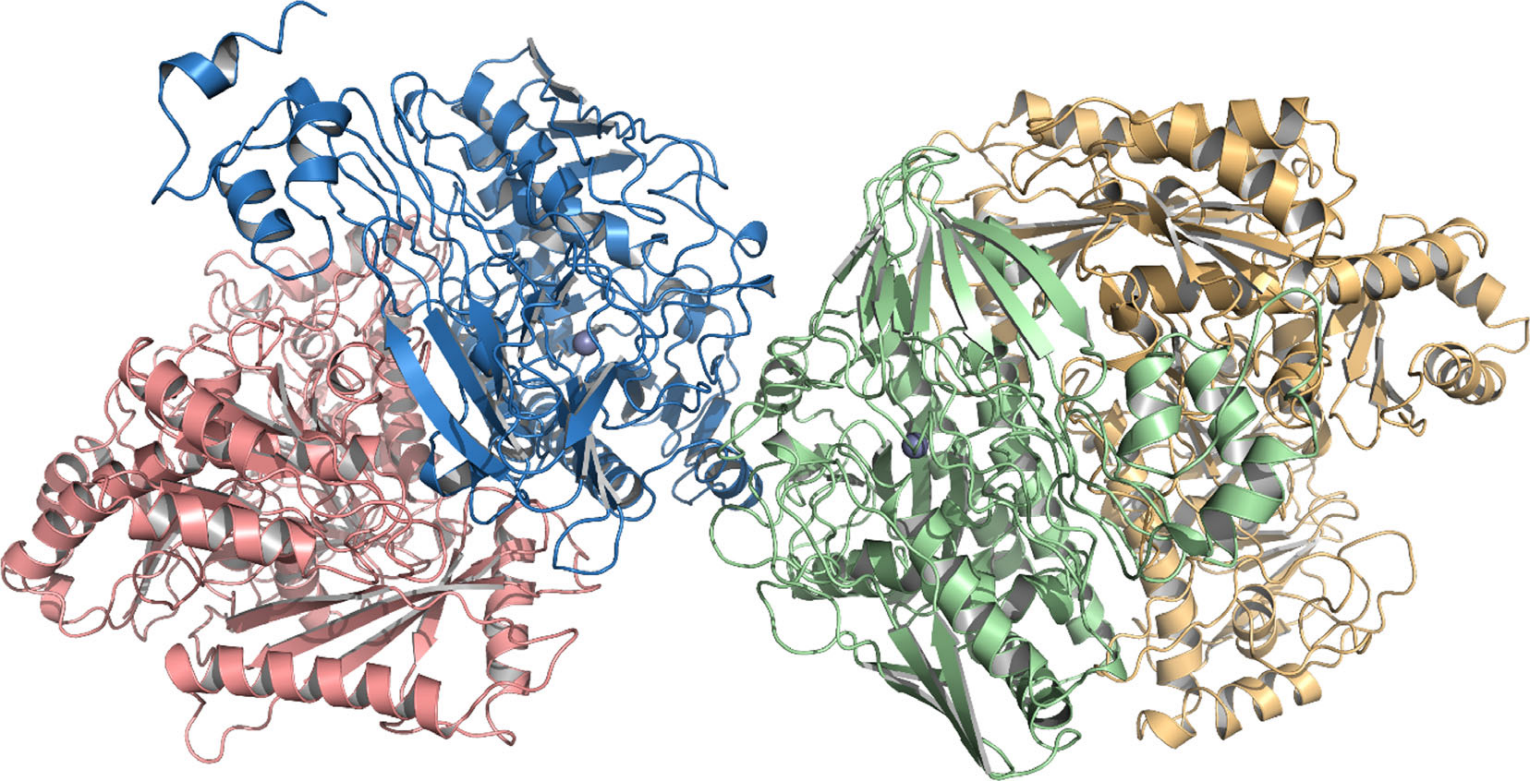
Crystal structure of caprolactamase tetramer. Salmon CapA, blue CapB, green CapB, yellow CapA. The assembly is (αβ)_2_. The metals are depicted as gray spheres [Color figure can be viewed at wileyonlinelibrary.com]

The structure shows a (αβ)_2_ heterotetrameric assembly. The final model thus consists of two CapB subunits (residues 1‐580) and two CapA subunits (residues 6‐696). The CapB subunits form a dimeric core with an interface area of ca. 1350 Å^2^ (Pisa server). Each CapB subunit interacts with a CapA subunit on the opposite side of the CapB‐CapB dimer interface, with a buried surface area of ca. 3000 Å^2^. The heterotetramer CapABBA has dimensions of ca. 70 Å × 80 Å × 170 Å (Figure 7). The elongated shape of the assembly observed in this crystal structure is in agreement with the results of dynamic light scattering (DLS) measurements with purified CapAB. Here, a homogeneous protein species was found with a hydrodynamic radius of 7.8 nm (Figure 4C), which corresponds well to the largest dimension of 17 nm found in the X‐ray structure of the tetramer. Due to the elongated structure the apparent molecular weight of the protein was overestimated at 412 kDa in DLS measurements; the mass of the tetrameric CapAB enzyme is 278 kDa.

The dimerization of the two CapB subunits is structurally similar to the interactions seen in the crystal structures of *Xa*Ac and *Aa*Apc. Polar as well as hydrophobic subunit interactions are observed. The YASARA hybrid model of CapAB predicted well the position of interface between the CapA and CapB despite the rotation between homologous subunits.^49^ However, the subunits in the YASARA model were ca. 2 Å closer to each other compared to the crystal structure, indicating despite the modest resolution the crystal structure identified clear flaws in the homology model.

### 2.6 Structure of CapA

**FIGURE 8:**
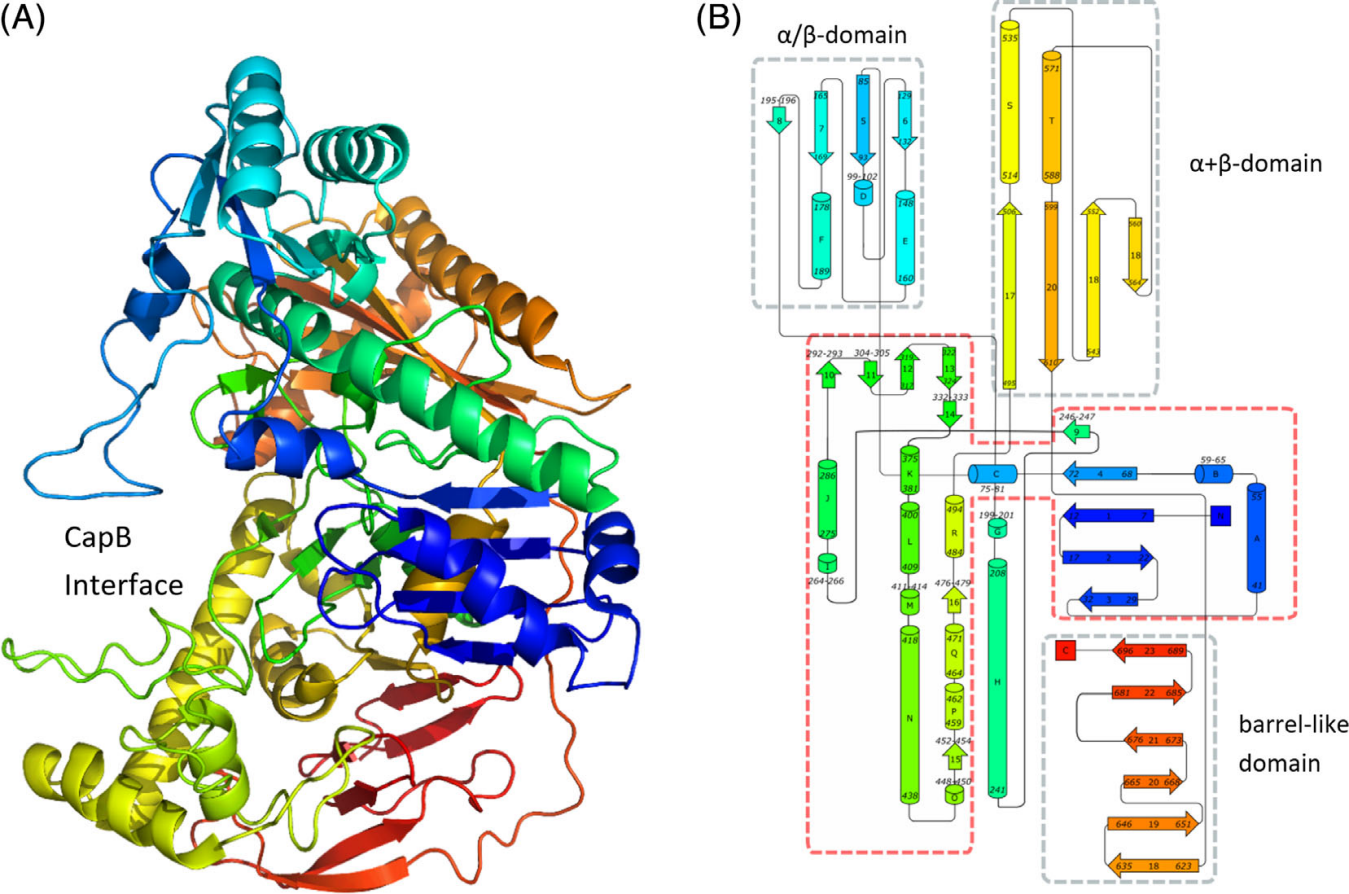
The CapA fold. A, Cartoon representation of CapA in rainbow depiction (N‐terminus blue, C‐terminus red) created with PyMol.^50^ B, Topology of the CapA fold. α‐helices are named by alphabetic letters and β‐strands are numbered. Diagram created on the Pro‐origami server^80^ and adjusted with the Inkscape graphics editor. The ASKHA ATP binding domain (red outline) is flanked by three additional domains, an α/β‐domain which also includes the long loops G and H (199‐241), an α + β‐domain and a C‐terminal barrel‐like domain (gray outline) [Color figure can be viewed at wileyonlinelibrary.com]

The CapA subunit comprises 20 α‐helices and 26 β‐strands involved in 6 β‐sheets (Figure 8) and is composed of an ATPase module common to acetate and sugar kinase/heat shock cognate/actin (ASKHA) superfamily proteins^52^ (residues 1‐84 and 242‐494) with an insertion of an α/β‐domain (res. 85‐241). The CapA structure is continued with an α + β‐domain (res. 495‐610) containing a disulfide bridge Cys545‐Cys605, and via a linking β‐strand to the C‐terminal barrel‐like domain (res. 623‐696). Dimerization with CapB occurs through interactions with a long helix (helix N, res. 418‐438) and an omega loop (res. 553‐560) of the α + β‐domain.

The structure supports the validity of the sequence‐based conclusion that CapA resembles the α and α′ subunits of acetophenone carboxylase from *A. aromaticum* and the corresponding large subunit of acetone carboxylase from *X. autotrophicus*. The structure of CapA is most similar to the ligand‐free state of the homologous acetophenone carboxylase subunit *Aa*Apcα (5L9WB 33% sequence identity and RMSD of 2.1 Å). The similarity to the *Aa*Apcα′ subunit is smaller (5L9Wb 20% id. and RMSD of 3.3 Å), which is in a closed, ADP‐bound state.^15^ Binding of ligands to acetone carboxylase also results in rearrangements in the β subunits.^14^ The *Xa*Acβ subunits containing AMP + 2 Mg^2+^ (5 M45), AMP + SO_4_ (5SVB) or SO_4_ (5SVC) show different conformations with RMSDs up to 2.8 Å. Therefore, it can be expected that ligand‐induced flexibility exists in the structures of the CapA according to the observations with the homologs.

The YASARA hybrid model of CapA has an RMSD of 2.2 Å with the crystal structure similar with *Aa*Apcα (5L9WB RMSD of 2.1 Å). The largest differences are observed in β‐strands 18 and 19 of the barrel like domain, loops connecting the secondary structures elements and the loop between α‐helix D and β‐strand 6 at the interface with CapB.

Inspection of structural alignments between CapA and the *Xa*Ac and *Aa*Apc subunits revealed a conserved (putative) ATP binding site in *Pj*CapAB. No ATP or ADP was observed in CapAB. Most of the CapA homologs have an aromatic amino acid residue stacking on the adenine ring: Phe405 in *Xa*Acβ, Phe368 in *Aa*Apcα′, and Tyr406 in *Aa*Apcα. The CapA subunit has two leucine residues at that position (Leu390, Leu391) (Figure 2) indicating fewer hydrophobic interactions. Non‐aromatic hydrophobic interaction is also observed in related proteins, for example, by side chains of arginine residues in the Hsp70‐x chaperone nucleotide binding domain (PDB code 6RZQ). There is no adenine binding motif in CapA.

Similar to AMP/ADP binding in homologous structures, the α‐phosphate (AMP) in CapA is probably bound via an Mg^2+^ ion to Asp18 and the backbone oxygens of Thr15, Phe16 and Gly298, and to the amide of the strictly conserved Lys32 (Figure 2). This α‐phosphate could also be bound via another Mg^2+^ ion to the carboxylate of Asp295, which will also have interaction to the second phosphate (ADP). This β phosphate and the γ phosphate (ATP) can also be bound via the same Mg^2+^ ions to the carboxylate atoms of Asp295 and Asp11 or side chains of Thr299 and Thr15.

The substrate binding site where the caprolactam will be phosphorylated should be close to γ‐phosphate binding site (ATP). It will be surrounded by Asp11 (conserved), Asn269, Pro272 (conserved) and Asp302 (conserved). There is no positively charged Lys or a His close to this site in CapA. In the absence of high resolution structures with bound substrate, the groups involved in enol tautomer formation and substrate phosphorylation remain unclear.

### 2.7 Architecture of CapB

**FIGURE 9:**
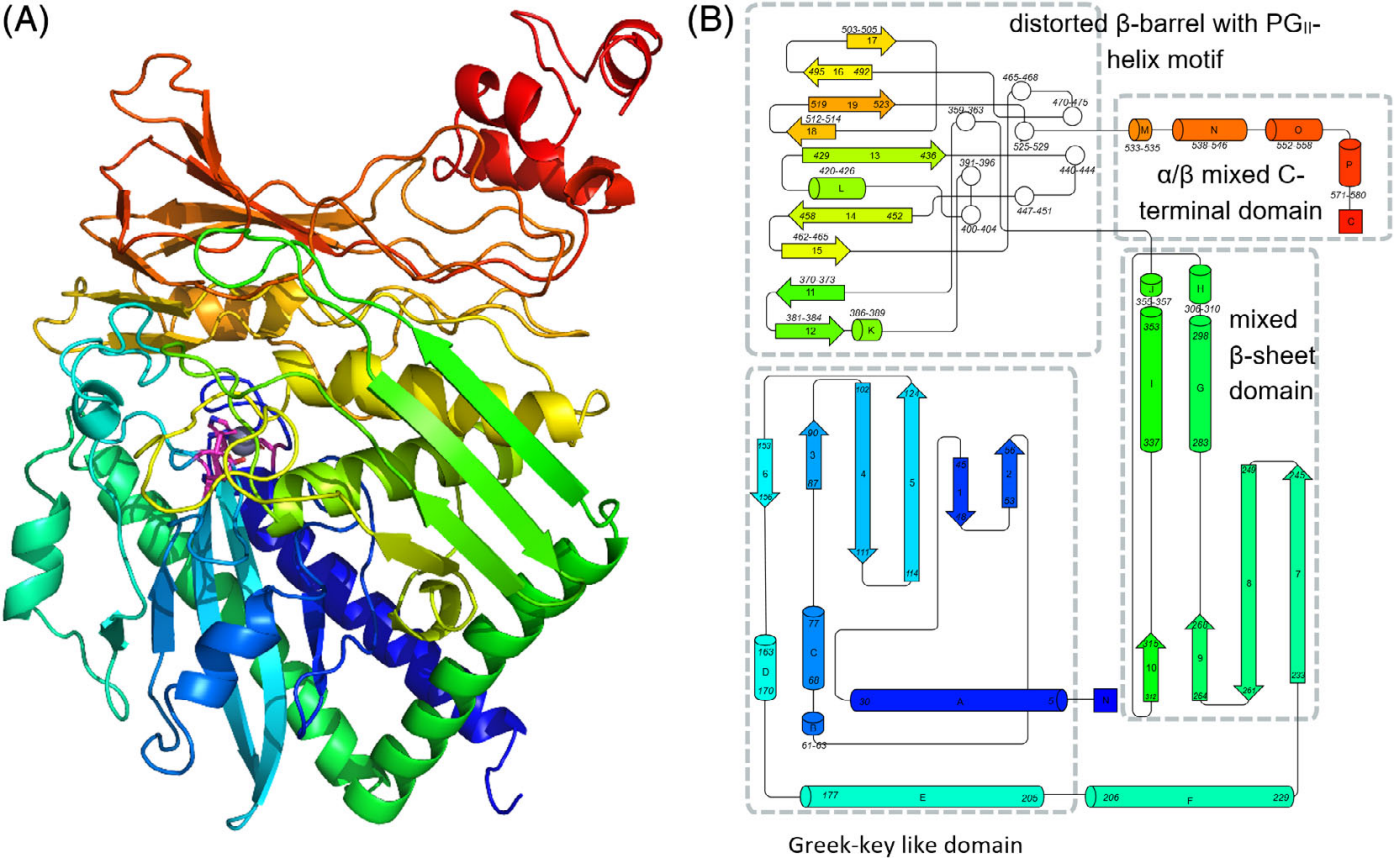
The CapB fold. A, Cartoon representation of CapB in rainbow depiction created with PyMol.^50^ The interface to CapA is at the back of the CapB molecule. Ligands Asp41, His99, Asp102, and His124 of the Zn^2+^ ion (gray) are colored in magenta; B, Topology of the main four domains in CapB. The N‐terminal Greek‐key like domain is followed by a mixed β‐sheet domain and a distorted barrel domain including the unusual PGII‐helix motif (white circles). A small mixed α/β domain is located at the C‐terminal end. The diagram was created on the Pro‐origami server^80^ and adjusted with the Inkscape graphics editor [Color figure can be viewed at wileyonlinelibrary.com]

The CapB subunit contains 11 long and 5 short α‐helices and 19 β‐strands involved in 5 β‐sheets (Figure 9). Its core structure is similar to that of the α subunit of acetone carboxylase (*Xa*Acα, RMSD 2.3 Å with 580 matched residues) and to the β subunit of acetophenone carboxylase (*Aa*Apcβ RMSD of 1.8 Å on 580 residues). The CapB structure commences with a Greek‐key like domain (residues 5‐205) from which the first and last long helices are involved in the dimer interface with CapA. A disulfide bridge is observed between Cys121 and Cys190. The structure continues via a linking helix to a mixed β‐sheet domain containing two α‐helices facing CapB (res. 233‐355), which is followed by a distorted barrel composed of two β‐sheets and containing the polyglycine type‐II‐like (PG_II_) helix motif (res. 358‐531) (Figure 3 & Figure 9B).^53^ CapB finishes with a small α/β mixed C‐terminal domain (res. 533‐581). Residues 129 to 174 of the Greek‐key like domain (sheet 6 and helix D) constitute the CapB‐CapB interface.

In agreement with the sequence analysis, CapB resembles the β subunit of *Aa*Apc and the corresponding subunit (α) of *Xa*Ac. CapB (581 residues) is smaller than the homologs *Aa*Apcβ (684 residues) and *Xa*Acα (776) subunits. In acetophenone carboxylase, the ca. 50 C‐terminal residues of the *Aa*Apcβ subunit embrace the *Aa*Apcγ subunit of which the function is unknown function and that is absent in caprolactamase. The C‐terminal arm of *Xa*Acα is ca. 90 residues long. It is involved in binding of the the *Xa*Acγ subunit and also enlarges the dimer interface (dimeric core of 3200 Å^2^). In addition, the N‐termini of *Aa*Apcβ and *Xa*Acα are 25 and 47 residues longer, respectively.

The YASARA hybrid model of CapB has an RMSD of 2.0 Å with the crystal structure similar with *Aa*Apcβ (5L9WB RMSD of 1.8 Å). The largest differences are observed in loops between β‐strands 15, 16 and 17 of the distorted β‐barrel domain (residues 473‐511). YASARA was not able to predict the correct folding of this domain which contains many glycine residues. Differences are also observed in a loop between β‐strand 9 and α‐helix D (res. 272‐278) of the mixed β‐sheet domain. Despite the modest resolution, the crystal structure provided a much better model of the structure, including positions of loop and interactions between domains and subunits.

Previous studies on the homologous ATP‐dependent carboxylases^41^, ^54^ suggest the presence of a divalent metal (Mn^2+^ or Zn^2+^) in the active site of the subunit homologous to CapB. In *Xa*Acα an Mn^2+^ ion is present in the active site and *Aa*Apcβ contains Zn or Fe. The corresponding metal‐binding residues in the CapB sequence are His99, Asp102, His124 (all three fully conserved in the decarboxylases, hydantoinases and oxoprolinases) and Asp41 (also conserved, but Glu89 in *Xa*Ac). The crystal structure of CapAB shows a metal at the expected site in CapB (Figure 9), but the nature of the ion is unknown. The electron density is too large for a Mg. There is no anomalous signal detected in the data, therefore a Mn^2+^ ion or a Fe^2+^ ion can be excluded (the anomalous scattering factors f” for Mn^2+^, Fe^2+^ and Zn^2+^ are 2.8, 3.2, and 0.68 e respectively at the data collection wavelength of 1.54 Å). However, Zn^2+^ has a negligible anomalous signal and was therefore tentatively modeled. Zn is also required by an ATP‐independent D‐hydantoinase^55^ and was modeled in the active site of a D‐hydantoinase 3‐D structure containing a binuclear metal site.^56^

The structure of of *Xa*Ac was determined in multiple conformational states. In 5SVB Glu89 is coordinating the metal, while in 5M45 and 5SVC the side chain of Glu89 is displaced indicating different conformations during the reaction. The metal‐bound state of Glu89 is observed with AMP bound and is allowing access of the phosphorylated intermediates to the Mn^2+^ active site.^49^ In CapB the equivalent Asp41 is a ligand of the metal and refines to this state. Refining the CapBA structure with a starting model containing an Asp41 in a displaced state always resulted in a shift of Asp41 to the metal‐bound state.

In CapB a cavity is located next to the metal binding site. This putative active site pocket has a surface area of 149 Å^2^ and a volume of 66.3 Å^3^. It is very hydrophobic and flanked by Leu38, Ile60, Ile62, Tyr136, Thr336, Met339, Ala365, Ile368, Val386, Ser389, Pro408, Leu415, Phe468, and a vicinal disulfide Cys410‐Cys411, providing a hydrophobic platform that possibly could bind caprolactam (Figure 3, Figure 11A & Figure S3). A disulfide bond between sequence‐adjacent cysteines is known as a vicinal disulfide bridge and in CapB it is of type T(rans)x. Disulfide groups from vicinal cysteines can bind to sugars or other rings.^57^ The disulfide is conserved in the *P. putida* lactamase (Table 1), but not in hydantoinases, 5‐oxoprolinases and the carboxylases. In *Xa*Ac this binding pocket is much smaller (33.4 Å^2^ area, 8.4 Å^3^ volume). There it is mainly constricted by three tryptophan residues (401, 438, and 479), Phe456, Leu108 and Met187, still leaving space for accommodating the smaller product acetoacetate. In *Aa*Apcβ this binding site is slightly smaller than in CapB and is also of hydrophobic nature (69.7 Å^2^ area and 26.6 Å^3^ volume).

### 2.8 Substrate channel

**FIGURE 10:**
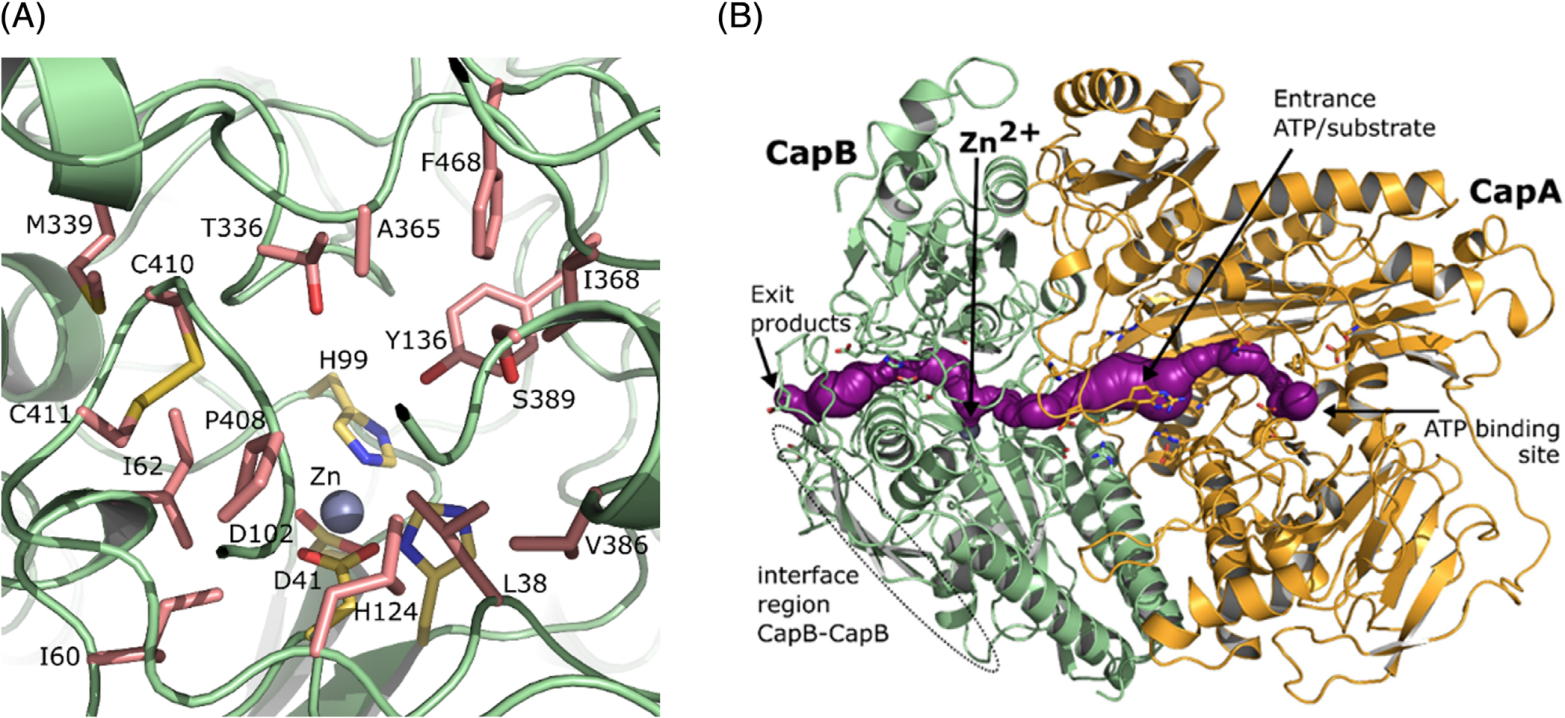
CapB active site. A, CapB pocket, surrounded by residues (salmon) Leu38, Ile60, Ile62, Tyr136, Thr336, Met339, Ala365, Ile368, Val386, Ser389, Pro408, Leu415, Phe468, and a vicinal disulfide Cys410‐Cys411. The metal (modeled as Zn^2+^) is depicted with a gray sphere. Ligands Asp41, His99, Asp102 and His124 of the Zn^2+^ ion are colored yellow. B, Possible intramolecular tunnel between CapA and CapB. The ATP binding site in CapA is connected to the metal binding site in CapB by a 40 Å long intramolecular tunnel. The charged loop in CapA (106 HKEDGHRYDATY 118) is marked with an arrow which indicates the entrance of ATP/substrate. Color coding as in panel A [Color figure can be viewed at wileyonlinelibrary.com]

The structural similarities between CapAB and carboxylases suggest that caprolactam and/or bicarbonate become phosphorylated by ATP in the CapA subunit, followed by movement to the CapB subunit for metal‐catalyzed lactam ring opening. In *Xa*Ac a substrate channel is proposed to connect the *Xa*Acβ and the *Xa*Acα subunits in which Glu89 shifts to a Mn^2+^‐coordinating position when the interior channel opens (PDB 5SVB).^14^ In CapB the corresponding Asp41 is already in a position where it coordinates the Zn^2+^ atom. This is comparable to the conformation of Asp65 in *Aa*Apcβ (PDB 5L9W). This metal‐coordinating conformation of Asp41 in CapB opens a long channel in the CapAB structure. A phosphorylated substrate formed in the ATP‐binding site of CapA can move through this channel, which is flanked by Asp302 (Asp315 in *Aa*Apc [5L9W]), Gln330 (Gln344), Arg112 (Gln116), His111 (absent in *Aa*Apc) and Arg439 (Glu455) to CapB Arg21 (Asp46 in Apc) and finally to Asp59 (Gly83) (Figure 10B). The phosphorylated substrate passes a charged loop in CapA (106 HKEDGHRYDATY 118). In *Aa*Apcα and *Xa*Acβ this loop is also present but in a different conformation, without contacting the γ‐domain. It is missing in *Aa*Apcα′ and probably shorter in 5‐oxoprolinase.

### 2.9 Active site mutants

To confirm the expected role of the putative ATP‐binding residues in CapA and of the observed metal‐binding residues in CapB in catalysis, we constructed a number of mutants. The two conserved aspartic acids located in the phosphate binding site 1 to 3 in subunit CapA (Asp11 and Asp295) likely play a role in ATP binding (vide supra). Of CapB we targeted the four residues (Asp41, His99, Asp101, and His124) involved in binding of the metal ion. These six residues were mutated to alanine and the mutant enzymes were produced and isolated by metal affinity chromatography as with wild‐type CapAB. Next, the enzymes were tested for their ability to convert caprolactam to 6‐ACA, which was measured by UPLC‐MS, and their thermal stability was also examined (Table 4).

**TABLE 4:** Properties of CapAB mutants

Enzyme variant	T_m_^app^ (°C)	Reaction time (h)	Conversion to 6‐ACA (%)
WT	56.0	1	100
CapA‐D11A	57.5	19	0.05
CapA‐D295A	55.0	17	0.4
CapB‐D41A	51.5	17	<0.015
CapB‐H99A	39.0	17	<0.015
CapB‐D102A	38.0	17	<0.015
CapB‐H124A	37.5	17	<0.015

*Note*: Reaction mixtures contained 50 mM ammonium bicarbonate buffer, pH 8, 2 mM caprolactam, 2 mM ATP, 5 mM MgCl_2_ and 5% glycerol.

The CapA phosphate site mutants retained their stability compared to the wild‐type enzyme but were able to convert only traces of caprolactam to 6‐ACA (Table 4). This small residual caprolactam hydrolysis activity found in CapA mutants was not observed in similar mutants of the 5‐oxoprolinase from *S. cerevisiae*.^30^ Because the activities were very low only end point measurements were taken to quantify the amount of 6‐ACA converted by these mutants. The four CapB metal binding site mutants completely lost the ability to convert caprolactam to 6‐ACA and also showed significantly reduced thermal stability (Table 4). These results confirm a role for the proposed ATP and metal binding sites in catalysis.

### 2.10 Mechanism of caprolactam hydrolysis

The sequence analysis, activity measurements, and comparison of X‐ray structures with other enzymes suggest possible catalytic mechanisms for *Pj*CapAB. The mechanism should account for two essential active sites connected by a tunnel, the uncoupling observed in the absence of caprolactam, and the essential role of bicarbonate both for ATP hydrolysis coupled to caprolactam hydrolysis and for uncoupled ATPase activity. The high uncoupling rate in the absence of caprolactam and in the presence of bicarbonate suggests that bicarbonate plays an active role in the catalytic cycle. Bicarbonate could be an allosteric activator triggering conformational changes but not taking part in the reaction. Conformational changes are of major importance in similar enzymes catalyzing ATP‐dependent substrate activation. However, in the related acetone and acetophenone carboxylases bicarbonate is a reactant that is activated to carboxyphosphate. This travels to the β subunit, together with the phosphorylated enol tautomer of the ketone acceptor, which is produced in the same α subunit as carboxyphosphate (acetone carboxylase) or in a different α′ subunit (acetophenone carboxylase). The close‐to‐one stoichiometric conversion of ATP and caprolactam during the caprolactamase reaction excludes that both bicarbonate and the organic substrate are phosphorylated separately, but not that activation of caprolactam involves carboxyphosphate as an intermediate. The formation of carboxyphosphate in the α subunit, distant from the caprolactam binding site, would be consistent with a high rate of ATP hydrolysis in the absence of caprolactam. A bicarbonate binding site like the one formed by two arginines and a glutamate in biotin carboxylase (PDB 4MV3) was not detected in CapA. The only positive group protruding into the active site is Lys40 which is conserved in the carboxylases, hydantoinases and lactamases but not in the oxoprolinases.

An examination of the expected position of the γ‐phosphate of ATP reveals a rather spacious site that could well accommodate bicarbonate and caprolactam. The most notable difference in charged residues around the region where substrate should bind are Asp302 in CapA, which corresponds to Lys281 in *Aa*Apcα′ of acetophenone carboxylase. Other differences are that two aromatic groups of *Aa*Apcα′ (Phe434 and Phe463) are missing in CapA. However, these differences neither rule out nor suggest that CapA phosphorylates bicarbonate.

From studies on pyruvate and propionyl‐CoA carboxylases as well as carbamoyl phosphate synthetases, it is known that carboxyphosphate is a crucial reaction intermediate generated by ATP dependent phosphorylation of bicarbonate.^58^, ^59^, ^60^ A distinct carboxylate binding site is present in biotin carboxylase (3G8C, 4MV9) where HCO_3_^−^ interacts with Glu, Lys, Asn, Arg^61^, ^62^ and in (N[5])‐carboxyaminoimidazole ribonucleotide synthase (3V4S), where HCO_3_^−^ is connected to Arg and Lys^63^. In both enzymes the formed carboxyphosphate does not diffuse from the active site but immediately transfers the carboxyl group to an acceptor. For these enzymes the role of bicarbonate is obvious since it forms a C–C or C–N bond. The role of bicarbonate in caprolactamases is less palpable and structural evidence for a bicarbonate binding site in CapA was not found. Yet, if carboxyphosphate is formed as an intermediate, it may transfer either the carboxylate moiety or the phosphate group to the iminol tautomer of caprolactam.

**FIGURE 11:**
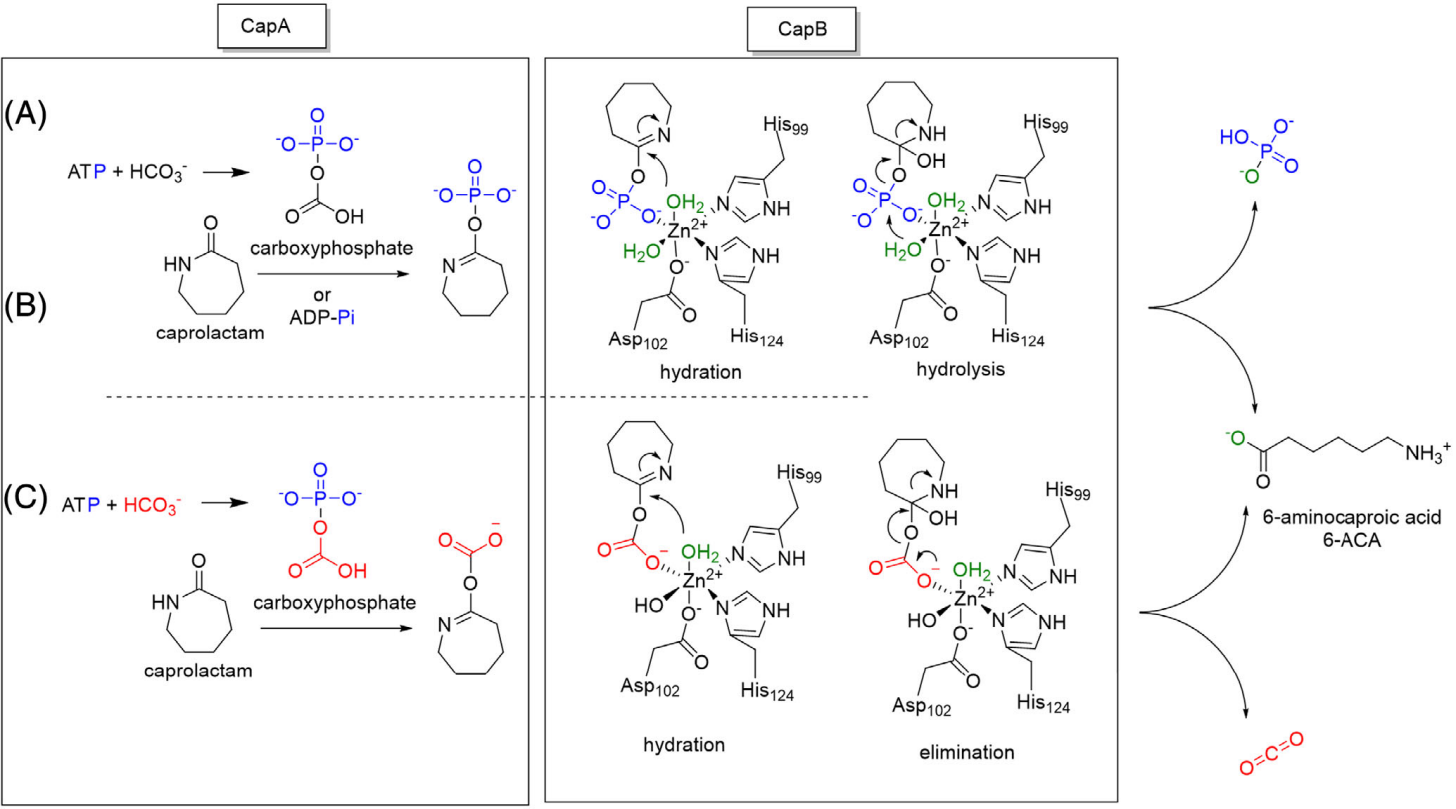
Proposed mechanisms for hydrolysis of caprolactam by *Pj*CapAB. A/B: Phosphorylation of caprolactam at the ATP‐binding site of the CapA subunit leading to a phosphorylated lactim intermediate either by direct phosphorylation by ATP, A or via the carboxyphosphate intermediate, B. After migration through a tunnel to CapB, hydrolysis of phosphocaprolactam occurs via coordinated water molecules releasing the product 6‐ACA and free phosphate. C, Carboxylation of caprolactam by carboxyphosphate followed by migration to CapB and subsequent elimination with involvement of coordinated water. Products CO_2_ (or bicarbonate) and 6‐ACA are released. Leaving group protonation is not shown [Color figure can be viewed at wileyonlinelibrary.com]

In view of the above, three different mechanisms can be considered (Figure 11). In mechanism A caprolactam acts as the direct acceptor in the substrate phosphorylation reaction and bicarbonate only functions as a non‐reacting activator, for example by introducing a conformational change that brings catalytic residues in proximity of the substrate. The –OH group of the lactim (iminol) tautomer of the substrate is phosphorylated by nucleophilic attack on the γ‐phosphate P of ATP in the CapA subunit, after which it moves and subjected to cleavage in CapB. This is in agreement with the proposed mechanism of oxoprolinases.^25^ Bicarbonate would be a non‐reacting activator. Bovine and human oxoprolinase are not reported to require bicarbonate for activity.^27^, ^28^ In mechanism B, caprolactam is also activated by phosphorylation of its lactim tautomer but with carboxyphosphate as a mediator in phosphoryl transfer from ATP to the lactim OH. In this scenario carboxyphosphate is formed from bicarbonate and ATP in CapA. Binding of bicarbonate could be comparable to the bicarbonate binding site in biotin carboxylases.^61^, ^62^ The third option, mechanism C, also involves carboxyphosphate but here caprolactam is not activated as the phosphorylated lactim, but as the carboxyester of the lactim. All three mechanisms involve formation of an activated caprolactim. This activated caprolactim intermediate would pass through the tunnel connecting CapA and CapB to the metal‐containing hydrolysis site in CapB. Structural inspection shows that the tunnel is sufficiently spacious (Figure 10B).

After migration through the tunnel to CapB, the activated caprolactim could be positioned close to the metal‐binding site next to two coordinated water molecules (Figure 11). We suggest the metal is a zinc, based on the absence of an anomalous signal in the crystallographic studies and in view of its presence in homologs, but activation by Ni cannot be ruled out, also because ATP‐dependent hydantoinase was promiscuous in its metal dependence.^34^ Metal‐assisted hydrolysis of the activated lactim requires one (in case of carboxylated lactim) or two (in case of phosphorylated lactim) water molecules, which can be activated by the metal in the CapB active site. Water deprotonation and amine protonation should facilitate the lactim ring opening. It likely includes a nucleophilic attack by water on the lactim carbon and protonation of the nitrogen of the amine that is formed. The only obvious group that could act as acid/base catalyst in phosphocaprolactim cleavage is Tyr136, which sticks into the active site of CapB. Through a nucleophilic attack by a coordinated water molecule a tetrahedral diol is formed. The second water molecule initiates the hydrolysis and 6‐ACA is and phosphate are released from the active site.

## 3 CONCLUSIONS

Caprolactamase belongs to a group of ATP‐dependent hydrolases of the oxoprolinase/ hydantoinase family of enzymes. Since the activities of these hydrolases are diverse (hydrolysis of lactams, hydantoins, oxoproline) but all act on cyclic amides, we propose the name ATP‐dependent lactamase family of enzymes. Furthermore, sequence similarities suggest that many genes annotated in sequence databases as *oplAB* in various *Pseudomonas* strains do not code for oxoprolinases but for lactamases acting on other substrates, for example, caprolactam or valerolactam. The crystal structure of caprolactamase shows an (α/β)_2_ tetramer, with the α subunits (CapA) responsible for ATP‐dependent substrate phosphorylation and the β subunits (CapB) for hydrolytic lactam ring opening. Multi‐sequence alignments and structural inspection suggest two distinct active sites, one with an actin‐like ATPase motif located in subunit CapA, which is conserved in a range of ATP utilizing enzymes from different functions.^64^ The second catalytic site is formed by a Zn‐binding site in CapB. The catalytic activity of caprolactamase was dependent not only on ATP but also on bicarbonate. The approximate equimolar formation of ADP and 6‐ACA suggests that only one ATP is used per round of caprolactam hydrolysis. The ATP dependence can be explained by an unfavorable position of the equilibrium, similar to that observed for oxoproline hydrolysis. The requirement for bicarbonate may be related to allosteric activation through conformational effects, but it is also conceivable that carboxyphosphate is formed and acts as a mediator in caprolactam activation, forming carboxy‐ or phospholactim. The dimeric structure and presence of a connecting tunnel suggest that the activated lactim moves from the CapA active site to the CapB active site without release to solvent. In the CapB site, activated substrate will undergo hydrolysis by water activated by bound metal. The results provide a basis for further studies aimed at identifying the nature of the activated lactim, the role of bicarbonate, the mechanism by which uncoupling is suppressed and the identification and role of catalytic groups.

## 4 MATERIALS AND METHODS

### 4.1 Isolation of His‐tagged caprolactamase

The previously constructed pET20b(+)‐based vector pET‐OP contains the *capAB* genes which were amplified from genomic DNA of *P. jessenii* GO3.^6^ To introduce a C‐terminal His‐tag on the α subunit called CapA, the insert and the vector were amplified using the PCR primer‐pairs prCapBA‐F (CCTCGGACTGATGATGTTCGCC) and prGib‐CapAHis‐R (GTGCTCGAGTGCGGCCGCTTAATGATGATGATGATGATGGCCG) for the insert and prGib‐CapAHis‐F (CGGCCATCATCATCATCATCATTAAGCGGCCGCACTCGAGCAC) with prCapBA‐R for the vector (GGCGAACATCATCAGTCCGAGG). The template DNA was digested with DpnI for 1 hour at 37°C and overlapping PCR products were purified (PCR purification kit, Qiagen) followed by assembly using ligation‐free Gibson cloning^65^ to obtain plasmid pET20b(+)‐CapBAHis. Subunit β or CapB stayed untagged. The vector was transformed into competent *E. coli* NEB10β cells. The introduction of the His‐tag and correct assembly were confirmed by DNA sequencing (Eurofins) and the plasmid was transformed into the expression strain *E. coli* C41(DE3).

For enzyme production, an overnight pre‐culture of *E. coli* C41(DE3) cells containing the expression vector was grown in LB medium with 100 μg mL^−1^ ampicillin and used to inoculate a main culture (1:100 dilution) in TB medium with ampicillin. Due to the autoinducing properties of TB medium, the culture was grown at 24°C for 24 hours and traces of lactose were sufficient to ensure expression of the *capAB* genes. The cells were harvested at 3000 x g at 4°C for 20 to 30 minutes, washed with AB buffer (50 mM ammonium bicarbonate‐NaOH buffer, pH 8.0, 10 mM MgCl_2_, 5% glycerol) and resuspended in AB buffer containing 1 mg mL^−1^ lysozyme (Sigma), an EDTA‐free protein inhibitor cocktail (cOmplete, Roche) and DNaseI (Sigma), and sonicated. Cell debris was removed by centrifugation for 45 minutes at 31000 x *g* and 4°C and the cell‐free extract was incubated with Ni‐NTA resin (Qiagen) for 1.5 hours at 4°C with head‐to‐tail rotation. The resin was washed extensively with AB buffer containing 20 mM imidazole and bound proteins were released with elution buffer (AB buffer with 300 mM imidazole). The protein sample was desalted using an EconoPac 10‐DG desalting column (Bio‐Rad) equilibrated with AB buffer to avoid aggregation. The enzyme was stored in AB buffer. Protein concentration and purity were determined by the Bradford assay using bovine serum albumin as the standard and by SDS‐PAGE (9%), respectively.

### 4.2 Site‐directed mutagenesis

To create CapAB mutants, site‐directed mutagenesis by Agilent's QuikChange protocol was performed for the active‐site positions Asp11 and Asp295 in CapA and Asp41, His99, Asp102, His124 in CapB, exchanging these functional residues by alanine. Primers were designed according to the requirements for QuikChange mutagenesis. PCR mixtures contained 1 μL template (50‐150 ng μL^−1^ DNA), 1 μL forward and reverse primer (10 μM each), 1.6% DMSO and 0.8 mM MgCl_2_ and were performed in a 25 μL reaction volume with PfuUltra II Hotstart 2× Master Mix (Agilent) in accordance with the provided thermocycling protocol. PCR template DNA was digested with DpnI and the PCR products were transformed to chemically competent *E. coli* NEB‐10β cells. The plasmids with successfully introduced mutations as verified by sequencing (Eurofins) were retransformed into competent *E. coli* C41(DE3) cells for enzyme production.

### 4.3 Caprolactamase activity assays

Standard reactions mixtures for caprolactamase assays contained 2 mM ATP, 2 mM caprolactam, 5 mM MgCl_2_ and 5% glycerol in a buffer of pH 8.0 and 90 μg of enzyme. Reactions were setup in 2 mL buffer. The buffer was varied between ammonium bicarbonate (AB buffer), Tris·HCl and Hepes·HCl, each at a concentration of 50 mM. Reactions were started by addition of enzyme and mixtures were incubated at 25°C. At different times samples of 80 μL were taken and mixed with 1 μL of formic acid after which precipitated protein was removed by centrifugation. The supernatants were used for analysis.

ATP and ADP were analyzed by HPLC on a Phenomenex Gemini C18 column (5 μm pore size, 4.6 × 250 mm) with a linear gradient (buffer A: 25 mM KH_2_PO_4_, 2.5% trimethylamine, 5% methanol, pH 6.5; buffer B: 25 mM KH_2_PO_4_, 2.5% trimethylamine, 50% methanol, pH 6.5). Retention times were 16 minutes for ADP and 17.5 minutes for ATP. The signal showed linear dependence in a range of 0 to 2 mM (Figure S4).

6‐ACA was analyzed by UPLC using separation on a Waters Acquity HSS T3 column (1.8 μm, 2.1 × 100 mm) operated with a linear gradient of water containing 0.1% formic acid (eluent A) to acetonitrile with 0.1% formic acid (eluent B) and with detection using an Acquity TQD mass spectrometer (Waters) operated in positive ion mode with multiple reaction monitoring for quantitative analysis. The fragment followed was m/z = 114 (6‐ACA M^+^‐H_2_O) (Figure S5).

### 4.4 Thermodynamic equilibrium of caprolactam hydrolysis

To predict the thermodynamic equilibrium for caprolactam hydrolysis, all species in Figure 6A need to be considered. We calculated the difference in Gibbs free energy between caprolactam and uncharged 6‐ACA (6‐ACA^0^) by quantum chemical methods, which is only accurate for species having the same charge.^66^ The equilibrium between the charged and uncharged forms of 6‐ACA was calculated from p*K*_A_ and p*K*_B_ values.

The change in Gibbs free energy upon hydrolytic ring opening of caprolactam to uncharged 6‐ACA (6‐ACA^0^) was calculated from the difference in formation energies of the three reactants $\left(\text{Δ}G^{\mathrm{Cap}+H_{2}O⇌6{\mathrm{ACA}}^{0}}\right)$. Quantum mechanical calculations of formation energies were performed under Gaussian09,^67^ using the computationally expensive but accurate CBS‐QB3 method with the water environment modeled using SMD.^44^, ^45^ The keywords were “opt freq cbs‐qb3 scrf=(solvent=water,smd)”. It was verified that no imaginary frequencies occurred in the final optimized structures. This gave $\text{Δ}G^{\mathrm{Cap}+H_{2}O⇌6{\mathrm{ACA}}^{0}}$ = 67 kJ/mol, indicating that formation of uncharged 6‐ACA would be disfavored when not accounting for charged species and 55 M water. When the effect of water concentration and all the relevant species (6ACA^tot^) are included, Equation 1 applies.

(1) $${\Delta G}_{0}^{\mathrm{Cap}⇌6{\mathrm{ACA}}^{\mathrm{tot}}}=\Delta G^{\mathrm{Cap}+H_{2}O⇌6{\mathrm{ACA}}^{0}}-RT\cdot \ln\left(\left[H_{2}O\right]\right)-RT\cdot \ln\left(\frac{\left[6{\mathrm{ACA}}^{\mathrm{tot}}\right]}{\left[6{\mathrm{ACA}}^{0}\right]}\right)$$

Assuming that the concentration of 6‐ACA^0^ is much smaller than that of charged species, and also that only one species is dominant at a certain pH,^20^ the relation between the total concentration of all species and uncharged 6‐ACA can be derived (Equation 2).

(2) $$\frac{\left[6{\mathrm{ACA}}^{\mathrm{tot}}\right]}{\left[{\mathrm{ACA}}^{o}\right]}=\left(10^{{\mathrm{pK}}_{B}^{-}+{\mathrm{pK}}_{A}^{+}-{\mathrm{pK}}_{A}^{0}-\mathrm{pH}}+10^{{\mathrm{pK}}_{B}^{-}-{\mathrm{pK}}_{A}^{0}}+10^{\mathrm{pH}-{\mathrm{pK}}_{A}^{0}}+1\right)\;$$

The assumptions reduce the distribution function of 6‐ACA to that of the single most dominant species. This causes a maximum error of RT·ln(2) (=1.7 kJ/mole), which would occur when two species have equal concentrations, i.e. when pH = *pK*_A_^+^ or pH = *pK*_B_^−^ (Figure 6).^20^ The microscopic *pK* values in Equation 2 are difficult to measure experimentally; therefore they were approximated in the usual manner from experimentally determined macroscopic constants.^20^ The *pK*_A_^0^ was estimated to be 4.8 from the similar non‐zwitterionic compound hexanoic acid. For p*K*_A_^+^ and p*K*_B_, the experimentally known values of 4.43 and 10.75 were used.^68^ Substituting Equation 2 into Equation 1 gives the pH‑dependent Gibbs free energy of caprolactam hydrolysis.

### 4.5 Crystallography of CapAB

Enzyme obtained by His‐tag isolation was further purified by gel filtration using a Superdex 200 HR10/30 column (GE Healthcare), equilibrated with 20 mM Tris·HCl buffer, pH 7.5, containing 150 mM NaCl, 10 mM MgCl_2_ and 2% glycerol. CapAB eluted at a molecular weight of ca. 320 kDa. CapAB fractions were pooled and concentrated to 5.1 mg mL^−1^ using an Ultracel‐30 K filter unit (Millipore). Dynamic light scattering (DLS) experiments were performed using a DynaPro MS800TC instrument (Wyatt Technology Corporation) at 20°C. DLS data were processed and analyzed with Dynamics software.

Sitting‐drop crystallization screening was performed using a Mosquito crystallization robot (TTP Labtech) in 96‐well MRC2 plates (Molecular Dimensions) with a protein concentration of 5.1 mg mL^−1^. Many commercially available screening solutions were tested. A small crystal, with a largest dimension of 0.1 mm, appeared after 1 month of incubation at 294 K in the Molecular Dimensions PACT screen condition G10; 0.02 M Na/K phosphate, 0.1 M Bis‐Tris propane, pH 7.5, and 20% PEG3350. This crystal could not be reproduced or improved although many optimization experiments were performed and numerous alternative crystallization conditions were investigated. Before data collection, the crystal was briefly soaked in a cryoprotectant solution, consisting of 25% glycerol, 25% PEG3350, 0.02 M Na,K phosphate and 0.1 M Bis‐Tris propane, pH 7.5. X‐ray diffraction data were collected on an in‐house MarDTB Goniostat System using Cu‐*Kα* radiation from a Bruker MicrostarH rotating‐anode generator equipped with HeliosMX mirrors. Intensity data were processed using XDS.^69^

The CapAB crystal belongs to the orthorhombic space group *P*2_1_2_1_2 with two dimers of 138 kDa (CapA is 75 kDa and CapB 63 kDa) in the asymmetric unit. The V_M_ is 2.6 Å^3^/Da with a solvent content of 52%.^70^ The structure of the CapAB was determined by the molecular replacement method using Phaser^71^ with an assembly of mixed model coordinates for CapB of *Xa*Acα (PDB code 5 M45:A)^14^ and *Aa*Apcβ (PDB code: 5L9W:A)^15^ generated by the FFAS server and the SCWRL algorithm.^72^ Phaser was able to find a dimer of CapB molecules. The position of the CapA molecules was calculated with an assembly of mixed models of *Xa*Acβ (PDB code 5SVB:B)^14^ and *Aa*Apcα (PDB code: 5L9W:B) and α′ (PDB code: 5L9W:b).^15^ Phaser determined the orientation of two CapAB dimers in the asymmetric unit with minimal clashes in the packing of the molecules and interpretable electron density.

Phenix Autobuild rebuild‐in‐place with non‐crystallographic symmetry was used for initial building and the model was further refined with Rosetta Phenix Refine and Phenix Refine.^71^, ^73^ Coot^74^ was used for manual rebuilding and map inspection. B‐factor sharpening was used for enhancement of the electron density.^75^ The quality of the model was analyzed with PDB_REDO^76^ and MolProbity.^77^ Composite omit electron‐density maps were used to validate the quality of the model.^45^ For comparison with the X‐ray structure, a 3D hybrid homology model of CapAB was generated using YASARA software (http://www.YASARA.org)^78^ with the crystal structures of *Aa*Apc (pdb 5L9W) and *Xa*Ac (5M45, 5SVB, and 5SVC) as templates.

The CAVER plugin for Pymol^50^, ^79^ was used to detect putative channels to the ATP binding site and substrate binding sites. For calculation of the characteristics of the channel, the CapB metal atom was set as a starting point. Channels were calculated with the following settings: minimum probe radius: 0.8 Å; shell depth: 10 Å; shell radius: 9 Å; clustering threshold: 3.5; number of approximating balls: 12; input atoms: 20 amino acids and the metal. Cavity volume were calculated with the program CastP^51^with a probe radius of 1.4 Å.

Atomic coordinates and experimental structure factor amplitudes were deposited in the Protein Data Bank accession number 6YRA.

## CONFLICT OF INTERESTS

The authors declare no potential conflict of interest.

## AUTHOR CONTRIBUTIONS

All authors designed experiments and/or contributed to the interpretation of the data. Antonija Marjanovic and Meintje S. de Vries cloned, mutated and expressed genes, isolated proteins and performed all characterization experiments. Henriëtte J. Rozeboom solved the crystal structure. Hein J. Wijma contributed energy calculations. Clemens Mayer, Hein J. Wijma and Marleen Otzen advised on the data interpretation. Antonija Marjanovic, Henriëtte J. Rozeboom and Dick B. Janssen wrote the manuscript.

### PEER REVIEW

The peer review history for this article is available at https://publons.com/publon/10.1002/prot.26082.

## Supporting information

**Appendix** S1. Supporting Information

## Data Availability

Atomic coordinates and experimental structure factor amplitudes are available from the Protein Data Bank (https://www.rcsb.org/) accession number 6YRA. Data that support the findings of this study are available in the supplementary material of this article. An expression clone for CapAB is available from the corresponding author upon request.
