# Supplementary material for: Catalytic and structural properties of ATP‐dependent caprolactamase from Pseudomonas jessenii
Source: Proteins. 2021 May 6;89(9):1079–98. doi: 10.1002/prot.26082 (PMC8453981; doi:10.1002/prot.26082)
Supplement: Supplementary file 1 — Appendix S1. Supporting Information [file PROT-89-1079-s001.pdf]

Supporting Information

Catalytic and structural properties of ATP-dependent caprolactamase from *Pseudomonas jessenii*

Antonija Marjanovic[1], Henriëtte J. Rozeboom[1], Meintje S. de Vries[1], Clemens Mayer[2], Marleen Otzen[1], Hein J. Wijma, and Dick B. Janssen[1]

[1] Biotransformation and Biocatalysis, Groningen Biomolecular Sciences and Biotechnology Institute (GBB), University of Groningen

[2] Biomolecular Chemistry and Catalysis, Stratingh Institute for Chemistry, University of Groningen.

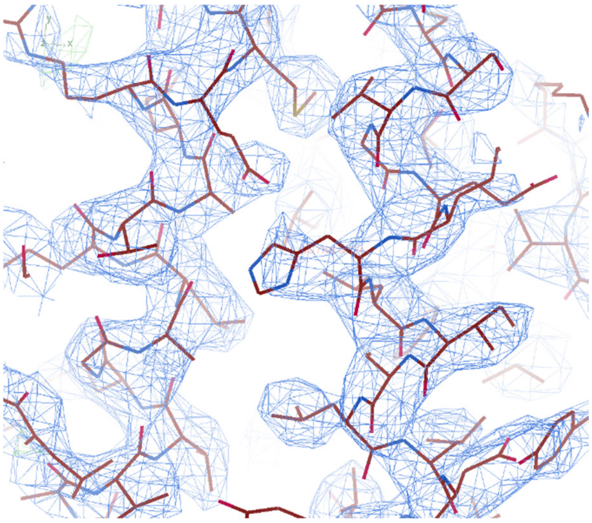

Figure S1: *2Fo-Fc* electron density map with B-factor sharpening of two parallel α-helices. Backbone and bulky side chains are clear. Electron density is shown in mesh, contoured at 1.0 sigma level; structural models are shown in stick.

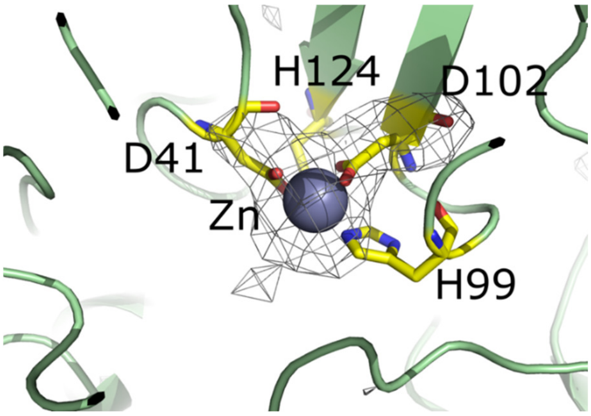

Figure S2. The active site of CapB with *Fo-Fc* omit electron density contoured at 3.0 σ. Omitted are Asp41, Asp102 and the zinc atom.

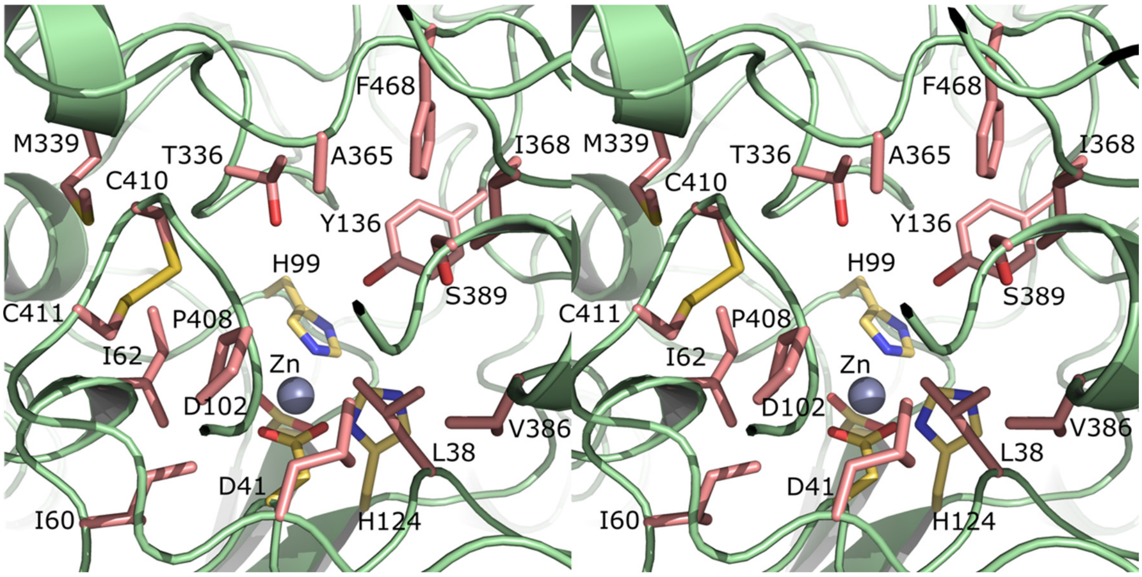

Figure S3: Stereo view of amino acids surrounding the substrate binding site of CapB. The CapB pocket is surrounded by residues (salmon) Leu38, Ile60, Ile62, Tyr136, Thr336, Met339, Ala365, Ile368, Val386, Ser389, Pro408, Leu415, Phe468, and the vicinal disulfide bridge Cys410-Cys411. The metal (modeled as Zn^{2+}) is depicted with a grey sphere. Ligands Asp41, His99, Asp102 and His124 of the Zn^{2+} ion are colored yellow. The image was constructed with PyMOL.

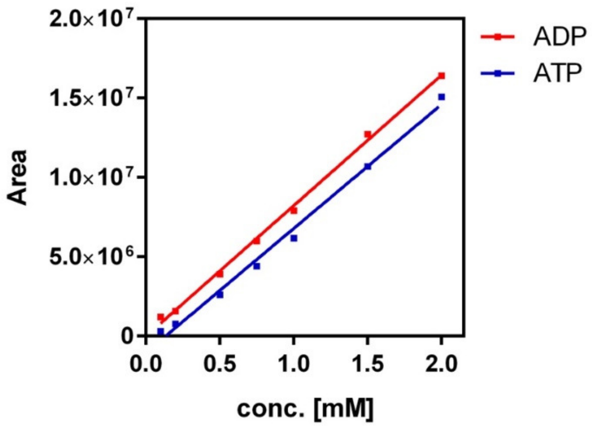

Figure S4: HPLC calibration curves for ADP and ATP (ATP: y = 7048396x; R^2 = 0.98 ADP: y = 8223685x; R^2 = 1.00).

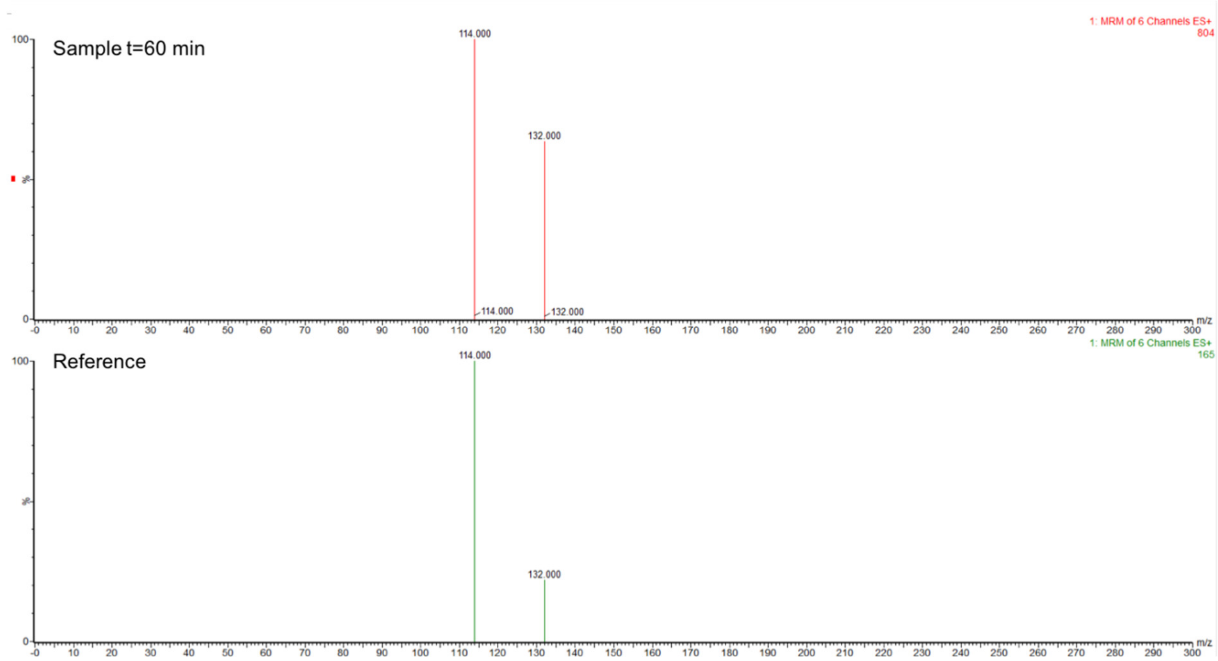

Figure S5: MS spectrum of 6-ACA. Top, reaction mixture after 60 min incubation; bottom reference sample. Electrospray impact, positive mode, MH^+= 132. See MassBank Record PR100222 for comparison.
